# Impact of acute kidney injury on the risk of mortality in patients with cirrhosis: a systematic review and meta-analysis

**DOI:** 10.1080/0886022X.2022.2142137

**Published:** 2022-11-15

**Authors:** Yunfeng Ning, Xiaoyue Zou, Jing Xu, Xiao Wang, Min Ding, Hulin Lu

**Affiliations:** a Department of Nephropathy, First Affiliated Hospital, Huzhou Teachers College, the First People’s Hospital of Huzhou, Huzhou, China; b Department of Emergency ICU, First Affiliated Hospital, Huzhou Teachers College, the First People’s Hospital of Huzhou, Huzhou, China

**Keywords:** Acute kidney injury, decompensated cirrhosis, terlipressin, mortality, complications, meta-analysis

## Abstract

**Objective:**

To compare the risk of mortality in patients with cirrhosis with and without the associated acute kidney injury (AKI).

**Methods:**

We performed a systematic search in the PubMed, Embase, and Scopus databases for observational studies that were done on patients with cirrhosis. Eligible studies reported AKI in patients with cirrhosis and compared mortality among patients with and without AKI. We used a random-effects model, using STATA version 16.0, for deriving pooled effect sizes that were reported as odds ratio (OR) with 95% confidence intervals (CIs).

**Results:**

Thirty-two studies were included. In patients with cirrhosis, AKI was significantly associated with higher in-hospital mortality (OR 5.92), and mortality at 30 days (OR 4.78), 90 days (OR 4.34), and at 1 year follow-up (OR 4.82) compared to patients without AKI.

**Conclusions:**

AKI is associated with an increased risk of mortality in patients with cirrhosis. Careful monitoring to identify the development of AKI and early prompt management is necessary.

## Introduction

Liver diseases in general and cirrhosis, in particular are among top 15 causes of death worldwide [1,2]. Cirrhosis is defined as the end stage of progressive fibrosis that irreversibly affects liver structure and function, and is associated with several clinical complications and reduced life expectancy [3]. The commonly documented causes of cirrhosis include viral hepatitis (Hepatitis B and C viruses), alcohol, and nonalcoholic steatohepatitis (NASH) [3]. Cirrhosis accounts for around 2% of mortality and 1.5% of DALYs (disability-adjusted life years) worldwide [1]. According to the Global Burden of Disease study, around 1.3 million deaths among females and 0.9 million deaths among males were related to cirrhosis in 2017 [4].

Advanced stages of cirrhosis are often accompanied by the impairment of renal function [5]. Acute kidney injury (AKI) in patients with cirrhosis may be caused by different factors. Some of these include decreased renal perfusion due to gastrointestinal bleeding, use of diuretics, diarrhea due to the use of lactulose or infections, and hepatorenal syndrome (HRS) characterized by renal vasoconstriction [5–7]. The pathophysiology of the AKI-HRS is related to disturbances in arterial circulation secondary to portal hypertension. The ensuing splanchnic pooling of blood reduces the effective circulating blood volume and, consequently, renal perfusion [8]. The diagnosis of AKI in patients with cirrhosis is challenging as there is fluid overload and high bilirubin levels that tend to interfere with creatinine assays [9].

There have been many studies documenting the incidence of AKI in patients with cirrhosis. The most recent systematic review that included 30 studies (*n* = 18,474 subjects) reported that 29% of patients with cirrhosis also had AKI [10]. However, this review had a substantial degree of heterogeneity, probably due to the differences in the diagnostic criteria that were used to define and stage AKI, such as Acute Kidney Injury Network (AKIN) guidelines, International Club of Ascites (ICA) 2015 criteria, Kidney Disease Improving Global Outcomes (KDIGO) criteria, Acute Dialysis Quality Initiative (ADQI) group guidelines and RIFLE (Risk, Injury, Failure, Loss of Kidney Function, and End-stage kidney disease) guidelines [10–15]. The current meta-analysis aimed to pool findings from published studies that documented the association between AKI and mortality in patients with cirrhosis.

## Materials and methods

### Search strategy

We used PubMed, Scopus, and Embase databases to systematically search for English language papers that were published until 1 April 2022. Medical subject heading (MeSH) terminology along with free text words was used. The search strategy is summarized in the supplementary document (Box 1). Only studies that were conducted in patients with a reliable diagnosis of cirrhosis and that compared risk of mortality among patients with and without AKI were considered for inclusion. The diagnosis of cirrhosis in the included studies was done using clinical, biochemical, radiological or histologic examination, or a combination of these methods. The outcome of interest was mortality. We followed the protocol that was registered at the International Prospective Registry of Systematic Reviews (PROSPERO; CRD42022324911), in adherence to the standard PRISMA (Preferred Reporting Items for Systematic Reviews and Meta-analyses) guidelines [16].

### Selection criteria and methods

After searching the databases and duplicate removal, studies were independently reviewed by two experts who screened titles and abstracts. Full texts of the relevant studies were carefully read, and disagreements were resolved by discussion. Bibliography sections of the selected studies were further searched for additional suitable studies.

#### Inclusion criteria

Studies that were observational in design and done in patients with cirrhosis; studies that reliably document the presence of AKI in patients with cirrhosis and compare risk of mortality in patients who developed AKI and those who did not.

#### Exclusion criteria

We excluded review articles and case reports. Studies that reported liver diseases other than diagnosed cirrhosis, and studies that compared risk of mortality without taking into account AKI diagnosis in patients with cirrhosis were excluded.

### Data extraction and quality assessment

A pretested data extraction sheet was used. Data extraction was done by two study authors independently and included study identifier i.e. name of the first author and year of publication; design of the study and place (country) of the conduct of the study; the important participant characteristics; definition of AKI and cirrhosis used; sample size in each of the two groups and relevant findings. The quality of the included studies was assessed using the Newcastle-Ottawa Quality Assessment Scale for observational studies [17].

### Statistical analysis

For this meta-analysis, STATA software, version 16.0 was used. Mortality-related outcome was reported as pooled odds ratio (OR) along with 95% confidence intervals (CIs). *I*^2^ was used as a measure to denote heterogeneity. The random effects model was used where *I*^2^ was more than 40% [18]. *p* < 0.05 was considered statistically significant. Publication bias was assessed by Egger’s test [19]. We conducted subgroup analysis based on the different diagnostic criteria of AKI adopted in the included studies, study design (i.e. prospective or retrospective) and stages of AKI. In instances of high heterogeneity, meta-regression was conducted to identify possible sources of heterogeneity.

## Results

### Selection of articles, study characteristics, and quality of included studies

**Figure 1.**
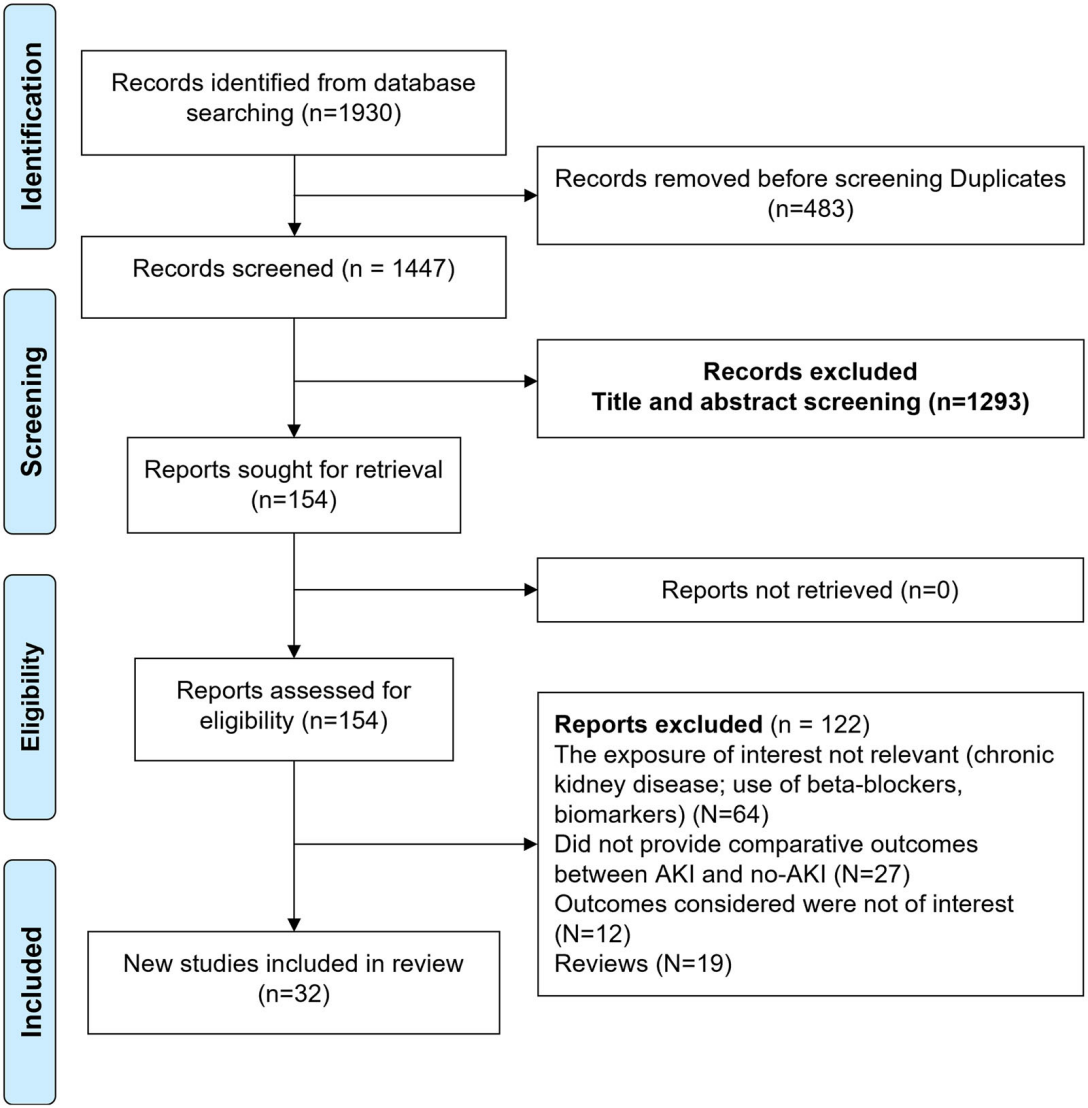
Selection process of the studies included in the review.

The systematic literature search identified a total of 1930 studies. Of them, 483 studies were removed as duplicates. Additional 1293 studies were excluded after the title and abstract screening. The full texts of the remaining 154 studies were reviewed and of them, 122 were excluded (Figure 1). Details of the 32 studies that were included in the final analysis [20–51] are summarized in Table 1. We did the assessment of inter-rater reliability after the first 400 records were screened and found it at 74.1%. The observed inter-rater reliability was mainly due to a lack of clarity and appropriate understanding of the inclusion and exclusion criteria among the two authors (YN and XZ). Therefore, a detailed discussion with the two authors involved in the screening and selection of studies was scheduled and led by the senior experienced author (XW). A clearly written inclusion and exclusion criteria were provided to both authors, and an overall inter-rater reliability of 94.7% was achieved.

**Table 1.** Characteristics of the studies included in the meta-analysis.

Author (year)	Study design; Country	Participant characteristics	Criteria used	Sample size	Key outcomes (AKI vs. no-AKI)
Leao et al. (2021) [20]	Prospective; Brazil	Patients with decompensated cirrhosis; mean age of 59.4 years; males (70%); alcoholic cirrhosis (38%) followed by Hepatitis C virus (38%)	Cirrhosis: histological aspects or on the interpretation of clinical, laboratory, radiological and endoscopic findings AKI: International Ascites Club; increase in sCr ≥0.3 mg/dL within 48 h or ≥50% from the baseline within the prior seven days	AKI (121); no-AKI (84)	Mortality (30-day): OR 9.88 (95% CI: 3.38, 28.9) Mortality (90-day): OR 9.79 (95% CI: 4.18, 22.9)
Makar et al. (2021) [21]	Retrospective; USA	Patients aged ≥18 years with a diagnosis of alcoholic cirrhosis; mean age of 59.4 years; female (38%); white race (67%)	The data on cirrhosis and AKI were retrieved from National Inpatient Sample (NIS) using the ICD codes. No detailing of the operational guidelines used to define both these clinical entities	AKI (6733); no-AKI (23,173)	Mortality (In-hospital): OR 8.09 (95% CI: 6.68, 9.79)
Moga et al. (2021) [22]	Prospective; France	Adult patients with cirrhosis; median age (∼62 years) and proportion male (∼73%) was similar in both groups; significant difference among AKI and non-AKI groups in the etiology of cirrhosis [alcoholic, 68% vs. 46.8% ; hepatitis C, 17% vs. 20.2%]; previous decompensation [ hepatic encephalopathy, 39.7% vs. 19%; ascites, 64.1% vs. 43.4%]	Cirrhosis: histological findings or a combination of clinical, biochemical, ultrasonographic, and endoscopic findings AKI: International Club of Ascites (ICA) 2015 criteria	AKI (78); no-AKI (327)	Mortality (in-hospital): OR 17.9 (95% CI: 7.3, 44.0) Mortality (30-day): OR 13.5 (95% CI: 6.6, 27.8) Mortality (90-day): OR 3.15 (95% CI: 1.33, 7.44)
Arora et al. (2020) [23]	Prospective; India	Patients above 18 years of age with decompensated cirrhosis; similar age of subjects in the two groups (48.8 years vs. 51.7 years); males (86.3%); 60% with alcoholic cirrhosis; hypertension in 8% and diabetes in 7%	Cirrhosis: history, physical examination, biochemical parameters, ultrasonography, and upper gastrointestinal endoscopy AKI: ICA-AKI criteria i.e., Increase in Serum Creatinine (SCr) ≥0.3 mg/dL within 48 h; or an increase in SCr ≥50% from baseline	AKI (71); no-AKI (104)	Mortality (90-day): OR 4.80 (95% CI: 2.12, 10.8)
Desai et al. (2020) [24]	Retrospective; USA	Mean age of 57 years; males (63%); white race (58%); alcoholic cirrhosis (52%); presence of chronic kidney disease (16%); diabetes (29%); hypertension (26%)	The data on cirrhosis and AKI were retrieved from clinical database using the ICD codes. No detailing of the operational guidelines used to define both these clinical entities	AKI (8,53,864); no-AKI (28,01,317)	Mortality (in-hospital): OR 3.75 (95% CI: 3.71, 3.79)
Kumar et al. (2020) [25]	Prospective; India	Patients with decompensated cirrhosis; mean age of 48 years; males (90%); alcoholic cirrhosis (65%) followed by Hepatitis B virus (26%)	Cirrhosis: clinical, biochemical, imaging, and endoscopic findings AKI: International Ascites Club- rise of serum creatinine ≥ 0.3 mg/dL within 48 h of admission or increase of serum creatinine ≥ 50% from stable baseline creatinine	AKI (82); no-AKI (40)	Mortality (30-day): OR 1.93 (95% CI: 1.26, 2.94)
Vaz et al. (2020) [26]	Prospective; Brazil	Patients with cirrhosis; mean age of 56.5 years; males (60%); alcoholic cirrhosis (36%); alcoholic with viral etiology (56%)	Cirrhosis: biopsy or combination of clinical, radiological, laboratory, and/or endoscopic findings AKI: ICA-AKI criteria- increase of sCr ≥ 0.3 mg/dL within 48 hours or a 50% increase from baseline sCr within the prior seven days.	AKI (89); no-AKI (65)	Mortality (In-hospital): OR 4.75 (95% CI: 1.10, 20.6)
Schacher et al. (2020) [27]	Retrospective; Brazil	Patients with cirrhosis; mean age of around 61 years; males (70%); alcoholic cirrhosis (25%); viral etiology, HCV (40%)	Cirrhosis: histological examination or on clinical, biochemical, endoscopic and imaging findings AKI: International Club of Ascites criteria- increase in sCr of ≥0.3 mg/dL from baseline within 48 hours or ≥50% from baseline within the prior seven days.	AKI (98); no-AKI (134)	Mortality (30-day): OR 11.9 (95%CI: 5.06, 28.4)
Sang Jo et al. (2019) [28]	Prospective; South Korea	Patients with decompensated cirrhosis; mean age (56 years vs. 59 years) and proportion male (66.7% vs. 74.2) was statistically similar in both groups; significant difference among AKI and non-AKI groups in the proportion with hypertension [20% vs. 22.7%] and diabetes [24.4% vs. 34.8%]	Cirrhosis: either liver biopsy or combined biochemical and imaging investigations AKI: kidney disease improving global outcomes (KDIGO) criteria	AKI (45); no-AKI (66)	Mortality (90-day): OR 2.52 (95% CI: 1.13, 5.64)
Jaques et al. (2019) [29]	Prospective; Geneva (Switzerland)	Patients with decompensated cirrhosis; mean age of 58 years; males (71%); proportion with hypertension (34% vs. 6%) and use of diuretics (70% vs. 39%)	Cirrhosis: combination of clinical, histological, biological and imaging data AKI: Acute Kidney Injury Network (AKIN) criteria	AKI (55); no-AKI (50)	Mortality (in-hospital): OR 6.0 (95% CI: 0.70, 51.7)
Marciano et al. (2017) [30]	Retrospective; Argentina	Patients with decompensated cirrhosis; mean age of around 62 years; 60% males; etiology (HCV- 23%; alcohol- 20%)	Cirrhosis: medical history + histology + clinical examination + biochemical and ultrasound/endoscopy examination AKI: kidney disease improving global outcomes (KDIGO) criteria	AKI (37); no-AKI (71)	Mortality (30-days): OR 3.91 (95% CI: 1.06, 14.4) Mortality (90-days): OR 2.26 (95% CI: 0.84, 6.06)
Nuthalapti et al. (2017) [31]	Retrospective; USA	Mean age of 57 years; males (63%); white race (20%)	Cirrhosis: clinical/ biochemical/radiological/ endoscopy AKI: Acute Kidney Injury Network (AKIN) criteria	AKI (51); no-AKI (233)	Mortality (30-days): OR 4.21 (95% CI: 1.99, 8.91) Mortality (90-days): OR 2.34 (95% CI: 1.16, 4.74)
Zhou et al. (2017) [32]	Retrospective; China	Mean age of 56 years; males (63%); etiology (HBV-70%; alcoholic- 9%)	Cirrhosis: clinical, biochemical, ultrasonographic and endoscopic findings AKI: kidney disease improving global outcomes (KDIGO) criteria	AKI (60); no-AKI (273)	Mortality (1yr): OR 16.9 (95% CI: 7.3, 39.6)
Hsieh et al. (2017) [33]	Retrospective; Taiwan	Mean age of 60 years; males (72%); diabetes (30%); etiology (alcohol- 20%; viral-64%)	Cirrhosis: biopsy of liver or the combination of clinical, biochemical, and radiological findings AKI: International Club of Ascites (ICA) criteria	AKI (46); no-AKI (67)	Mortality (90-day): OR 7.27 (95% CI: 2.44, 21.6)
Biyik et al. (2016) [34]	Retrospective; Turkey	Patients with cirrhosis; study sample with a mean age of 62 years; males (60%); no differences in baseline characteristics between two groups; common etiology of cirrhosis included HBV, HCV infection and cryptogenic cirrhosis	Cirrhosis: Combination of physical, biochemical, endoscopic and imaging investigations AKI: kidney disease improving global outcomes (KDIGO) criteria	AKI (108); no-AKI (169)	Mortality (in-hospital): OR 23.3 (95% CI: 8.02, 67.7)
Pan et al. (2016) [35]	Prospective; Taiwan	Mean age of 58 years and males (around 76%); etiology (majority alcohol; HBV or HCV)	Cirrhosis: medical history + histology + clinical examination + biochemical and ultrasound examination AKI: RIFLE (Risk, Injury, Failure, Loss of Kidney Function, and End-stage kidney disease), AKIN, and KDIGO guidelines	AKI (152); no-AKI (90)	Mortality (in-hospital): OR 5.25 (95% CI: 2.98, 9.27)
Shi et al. (2016) [36]	Prospective; China	Patients with HBV related acute-on-chronic liver disease; mean age of 45 years; males (85%); mean BMI of 23 kg/m2	AKI: kidney disease improving global outcomes (KDIGO) criteria	AKI (308); no-AKI (859)	Mortality (30-day): OR 1.87 (95% CI: 1.44, 2.43) Mortality (90-day): OR 4.47 (95% CI: 3.30, 6.05) Mortality (1 yr): OR 4.83 (95% CI: 3.50, 6.66)
Jindal et al. (2016) [37]	Retrospective; India	Mean age of 46 years; males (85%); etiology (alcoholic- 46%; viral- 38%)	Cirrhosis: clinical examination/imaging/endoscopy AKI: >50% increase in SCr level from baseline in less than 6 months; alternatively, increase of ≥0.3 mg/dl in less than 48 h or SCr more than1.5 mg/dl at admission in the absence of documented CKD (chronic kidney disease)	AKI (55); no-AKI (186)	Mortality (30-days): OR 3.19 (95% CI: 1.46, 6.93) Mortality (90-days): OR 2.86 (95% CI: 1.48, 5.55)
Tandon et al. (2016) [38]	Retrospective; Canada	Mean age of around 60 years; males (64%); co-associated diabetes (20%); previous myocardial infarction (around 10%)	Cirrhosis: defined using validated algorithm AKI: kidney disease improving global outcomes (KDIGO) criteria	AKI (1850); no-AKI (2883)	Mortality (30-day): OR 8.46 (95% CI: 7.20, 9.93) Mortality (90-day): OR 8.42 (95% CI: 7.18, 9.88)
Bucsics et al. (2015) [39]	Retrospective; Austria	Mean age of 55 years and males (around 67%); etiology (majority alcohol-59% and viral-16%)	Cirrhosis: biopsy/transient elastography/imaging/clinical Signs AKI: increase in sCr by ≥0.3 mg/dL or by ≥50% either from baseline	AKI (78); no-AKI (161)	Mortality (30-day): OR 3.36 (95% CI: 1.77, 6.37)
Angeli et al. (2015) [40]	Prospective; Spain	Patients with decompensated cirrhosis; mean age of 55 years; males (65%); etiology (alcoholic- 60%; HCV-13%); co-associated diabetes (9%)	Cirrhosis: not defined AKI: Acute Kidney Injury Network (AKIN) criteria	AKI (98); no-AKI (412)	Mortality (30-day): OR 5.34 (95% CI: 3.32, 8.60) Mortality (90-day): OR 3.67 (95% CI: 2.30, 5.84)
Choi et al. (2014) [41]	Retrospective; South Korea	Patients with cirrhosis; Male (74%); mean age of around57years; major cause of cirrhosis (alcohol-50%; HBV-31%)	Cirrhosis: Combination of physical, biochemical, endoscopic and imaging investigations AKI: Acute Kidney Injury Network (AKIN) guidelines	AKI (83); no-AKI (560)	Mortality (in-hospital): OR 1.35 (95% CI: 0.41, 4.44)
Araujo et al. (2014) [42]	Prospective cohort; Brazil	Mean age of subjects 57 years; males (63%); predominant etiology (67%); high Child-Pugh score (78%); mean MELD score of 19.5	Cirrhosis: clinical examination and/or biopsy based AKI: Acute Kidney Injury Network (AKIN) criteria	AKI (20); no-AKI (26)	Mortality (90 days): OR 6.85 (95% CI: 1.77, 26.5) Mortality (in-hospital): OR 25.0 (95% CI: 2.82, 221.7) Mortality (1 yr): OR 3.41 (95% CI: 1.58, 7.36)
Piano et al. (2013) [43]	Prospective; Italy	Mean age of 65 years; males (65%); etiology (alcohol- 34%; HCV- 40%); median Child-Pugh score of 9 and MELD score of 16	Cirrhosis: Combination of physical, biochemical, endoscopic and imaging investigations AKI: Acute Kidney Injury Network (AKIN) criteria and Conventional criteria	AKI (61); no-AKI (172)	Mortality (in-hospital): OR 8.26 (95% CI: 3.70, 18.5)
Scott et al. (2013) [44]	Prospective; United Kingdom	Mean age of 57 years; male (65%); alcoholic liver disease (78%); patients characteristics were similar among the two groups, except for diabetes prevalence which was higher in AKI group (29% vs. 11%)	Cirrhosis: Combination of physical, biochemical, endoscopic and imaging investigations AKI: Acute Kidney Injury Network (AKIN) criteria	AKI (110); no-AKI (52)	Mortality (in-hospital): OR 11.7 (95% CI: 2.68, 50.7)
Wong et al. (2013) [45]	Prospective; USA	Mean age of 56 years; males (56%); etiology (alcohol-31%; viral-26%)	Cirrhosis: combination of biochemical/imaging and endoscopic findings AKI: International Ascites Club and ADQI guidelines	AKI (166); no-AKI (171)	Mortality (30-day): OR 6.75 (95% CI: 3.45, 13.2)
Fagundes et al. (2013) [46]	Prospective; Spain	Mean age of 61 years; males (62%); diabetes (29%); hypertension (23%); etiology (alcohol- 41%; HCV-35%)	Cirrhosis: relevant data not provided AKI: Acute Kidney Injury Network (AKIN) criteria	AKI (177); no-AKI (198)	Mortality (90-day): OR 4.63 (95% CI: 2.75, 7.81)
Tsien et al. (2013) [47]	Prospective; Canada	Patients with decompensated cirrhosis; mean age of 56years; males (64%); etiology (>90% either alcoholic or viral)	Cirrhosis: either biopsy confirmed or combination of laboratory/ endoscopy/ imaging AKI: serum creatinine of ≥50% from baseline, or a rise of serum creatinine by ≥26.4 mmol/l in less than 48 hours	AKI (49); no-AKI (41)	Mortality (1yr): OR 7.80 (95% CI: 0.93, 65.3)
Hung et al. (2012) [48]	Retrospective; Taiwan	Patients with cirrhosis and spontaneous bacterial peritionitis; mean age of 60 years; males (70%); etiology (hepatocellular carcinoma-20%; alcohol-15%); diabetes mellitus (10%)	Cirrhosis and AKI: ICD-9-CM diagnosis	AKI (300); no-AKI (2292)	Mortality (30-day): OR 3.48 (95% CI: 2.71, 4.48) Mortality (1-yr): OR 2.27 (95% CI: 1.76, 2.94)
Warner et al. (2011) [49]	Retrospective; USA	Mean age of 53 years; ethnicity mainly African American and White; males (76%); etiology (alcohol- 38%; HCV- 45%); Mean Child-Pugh Score of 10 and MELD score of 25	Cirrhosis: Combination of clinical, imaging and pathological investigations AKI: Acute Kidney Injury Network (AKIN) criteria	AKI (107); no-AKI (19)	Mortality (in-hospital): OR 1.57 (95% CI: 0.71, 3.45)
Cholongitas et al. (2009) [50]	Prospective; United Kingdom	Patients with decompensated cirrhosis; mean age of 49 years and males (around 60%); etiology (alcohol- 65%; HBV or HCV- 17%)	Cirrhosis: not provided AKI: serum creatinine ≥ 300 mmol/l, urine output (<500 ml/ day), presence of hepatorenal syndrome or need for hemofiltration	AKI (128); no-AKI (184)	Mortality (in-hospital): OR 6.5 (95% CI: 3.35, 12.6)
Cheyron et al. (2005) [51]	Retrospective; France	Mean age of 56 years; males (70%); majority with alcoholic cirrhosis (72%); no statistical difference in the proportion of subjects with diabetes, hypertension, cardiac disease in the two groups	Cirrhosis: histologically confirmed and/or clinically diagnosed cirrhosis AKI: Acute Dialysis Quality Initiative (ADQI) group guidelines	AKI (73); no-AKI (113)	Mortality (in-hospital): OR 4.11 (95% CI: 2.20, 7.67)

All the included studies were observational. Of them, 17 studies were prospective and 15 studies were retrospective. Five studies were done in the USA, four in Brazil, three in India, and three in Taiwan. Two studies each were done in South Korea, China, the United Kingdom, Canada, Spain, and France. There was one study each from Switzerland, Turkey, Argentina, Austria, and Italy. There were differences in the studies in terms of AKI definition. Some of the commonly used diagnostic criteria were Acute Kidney Injury Network (AKIN) guidelines, International Club of Ascites (ICA) 2015 criteria, kidney disease improving global outcomes (KDIGO) criteria, Acute Dialysis Quality Initiative (ADQI) group guidelines, and RIFLE (Risk, Injury, Failure, Loss of Kidney Function, and End-stage kidney disease) guidelines (Table 1). The included studies mostly defined “cirrhosis” based on a combination of clinical examination, medical history, histology, biochemical, and imaging (ultrasound or endoscopy) examination (Table 1). In the included studies, a small sub-group of subjects with AKI had type 1 or type 2 hepatorenal syndrome (HRS). The proportion of patients with HRS in the included studies varied from 5% to 35%. As summarized in Supplementary Tables 1–3, all the included studies were of high to modest quality.

### AKI and risk of mortality

**Figure 2.**
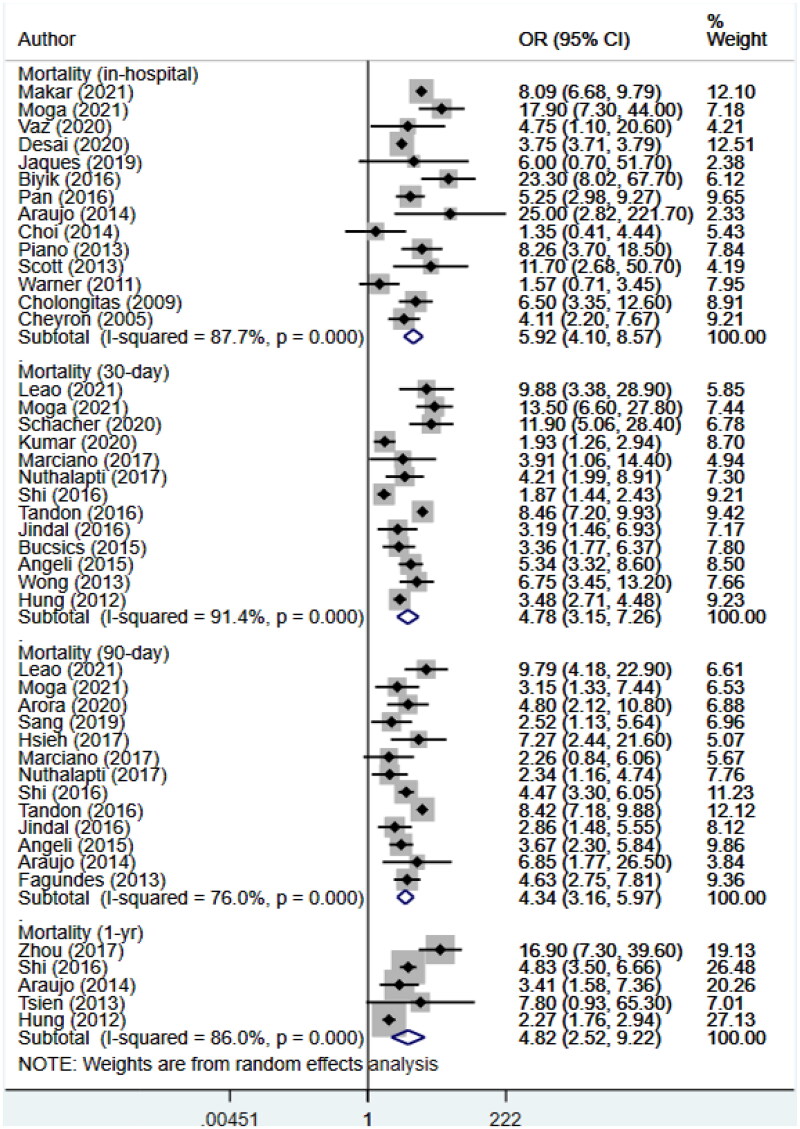
Association of acute kidney injury (AKI) with mortality within 1 year follow-up.

AKI was associated with significantly higher in-hospital mortality (OR 5.92, 95% CI: 4.10, 8.57; *N* = 14; *I*^2^ = 87.7%), mortality at 30 days (OR 4.78, 95% CI: 3.15, 7.26; *N* = 13; *I*^2^ = 91.4%), mortality at 90 days (OR 4.34, 95% CI: 3.16, 5.97; *N* = 13; *I*^2^ = 76%) and mortality at 1 year follow-up (OR 4.82, 95% CI: 2.52, 9.22; *N* = 5; *I*^2^ = 86%) (Figure 2), compared with patients without AKI. Visual inspection on funnel plots and Egger’s test showed no publication bias for the risk of mortality at different time points (*p* > 0.05) (Supplementary Figures 1–4).

### Findings of the subgroup analysis

For the risk of in-hospital mortality, subgroup analysis based on the different diagnostic criteria of AKI adopted by the studies showed increased risk in patients with AKI, compared to patients without AKI (Supplementary Figure 5). The 95% confidence intervals were wide due to the fewer number of studies. An increased risk of 30-day mortality (ICA: OR 5.4, 95% CI: 2.6, 11.1; *N* = 6; *I*^2^ = 84.3%; KDIGO: OR 4.0, 95% CI: 1.2, 13.7; *N* = 3; *I*^2^ = 97.9%; AKIN: OR 5.0, 95% CI: 3.3, 7.5; *N* = 2; *I*^2^ = 0.0%) and 90-day mortality (ICA: OR 4.7, 95% CI: 2.9, 7.6; *N* = 5; *I*^2^ = 37.9%; KDIGO: OR 4.4, 95% CI: 2.4, 7.8; *N* = 4; *I*^2^ = 87.9%; AKIN: OR 3.8, 95% CI: 2.8, 5.2; *N* = 4; *I*^2^ = 3.9%) was noted across all the adopted diagnostic criteria of AKI (Supplementary Figures 6 and 7). For mortality at 1 year follow-up, an increased risk was noted for all diagnostic criteria of AKI but the effect sizes were wide due to very few studies (Supplementary Figure 8). On subgroup analysis based on the stages of AKI (i.e. stages 1–3), we found that the risk of mortality in each of the stages was higher, compared to no AKI, at all-time points i.e. in-hospital, 30-days, 90-days and at 1 year follow-up and at each of these time-points, the magnitude of risk increased with the increasing stage of AKI (Supplementary Figures 9–12).

**Figure 3.**
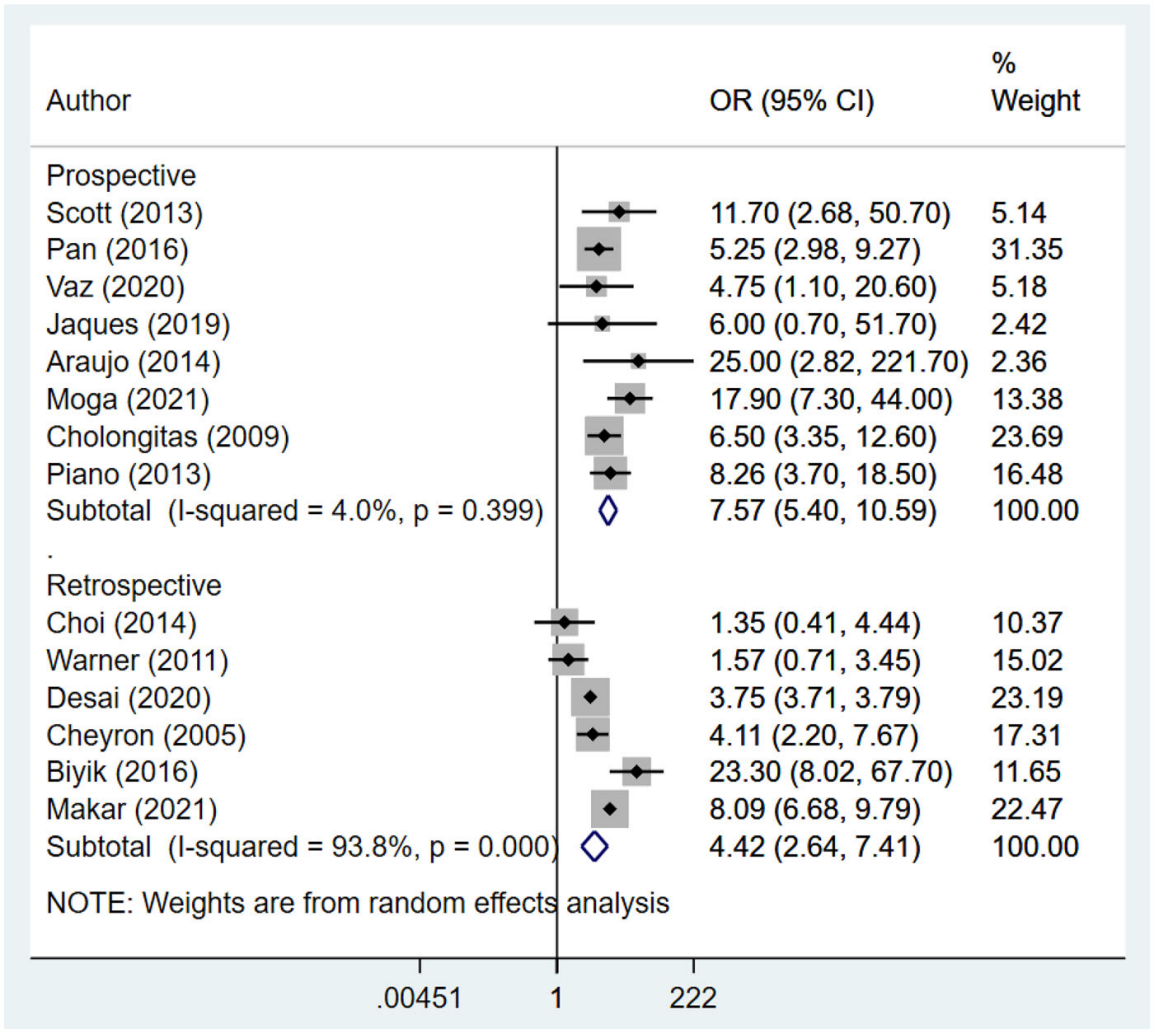
Subgroup analysis for association of acute kidney injury (AKI) with in-hospital mortality based on design of included studies.

**Figure 4.**
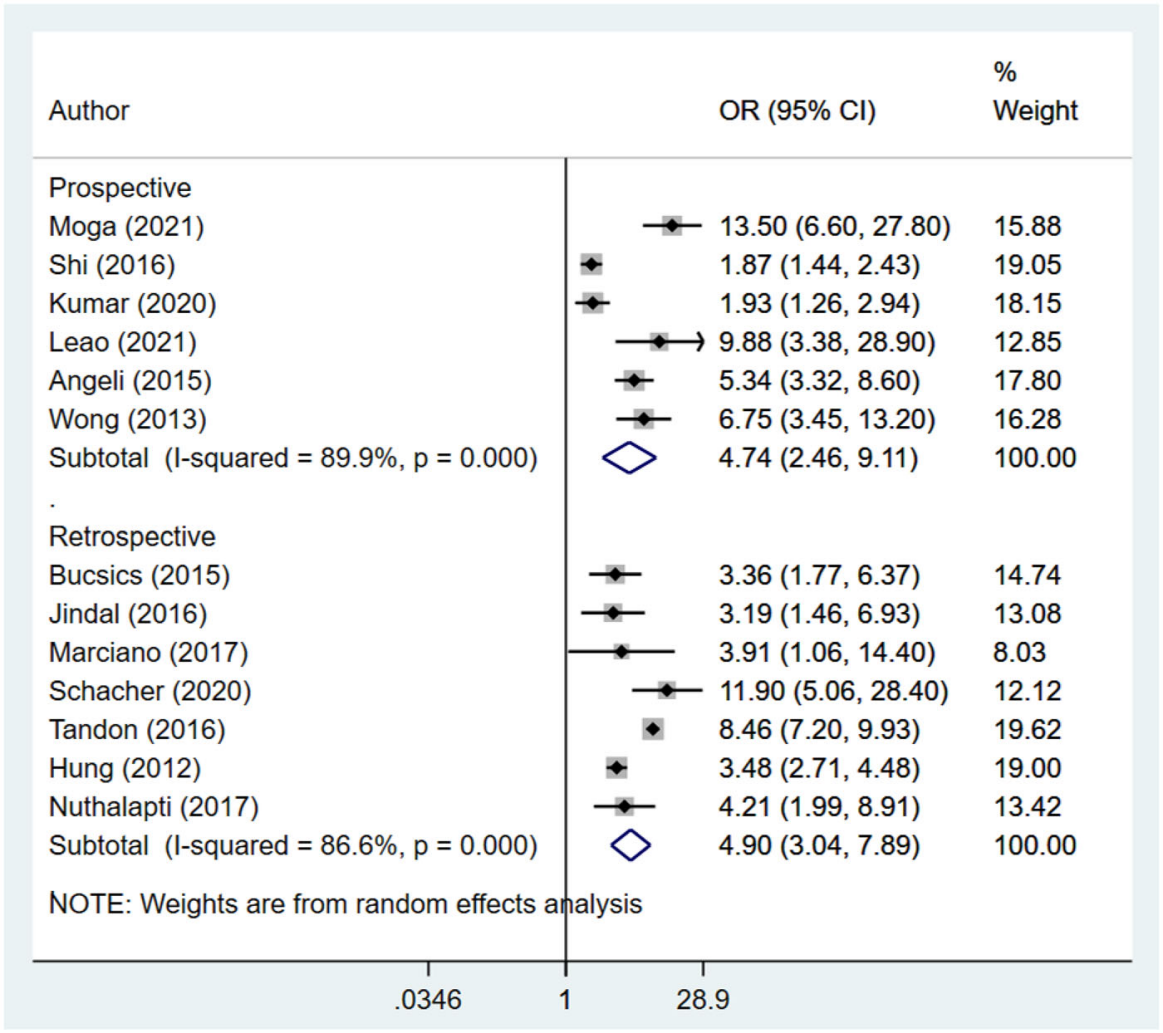
Subgroup analysis for association of acute kidney injury (AKI) with 30-day mortality based on design of included studies.

**Figure 5.**
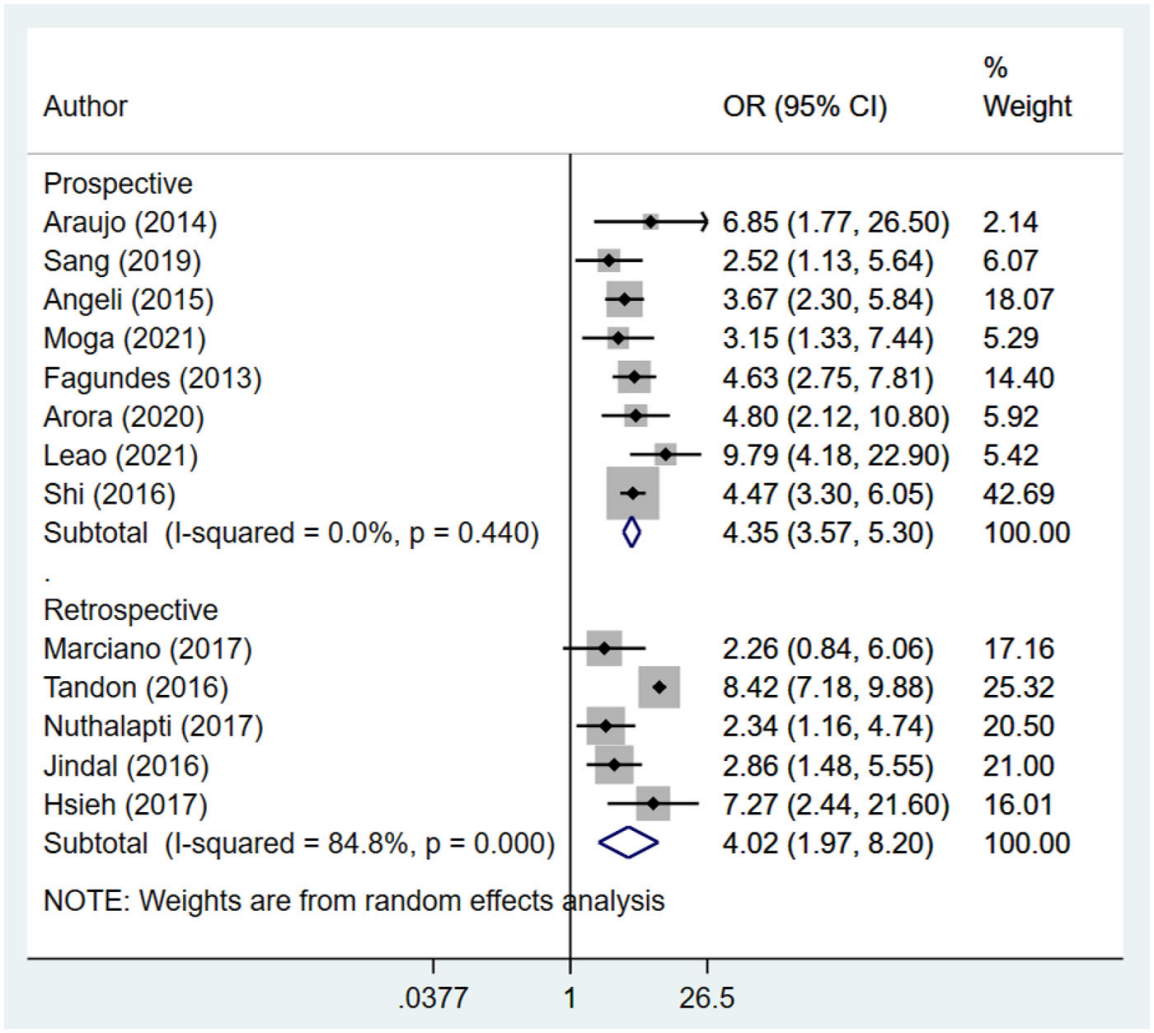
Subgroup analysis for association of acute kidney injury (AKI) with 90-day mortality based on design of included studies.

**Figure 6.**
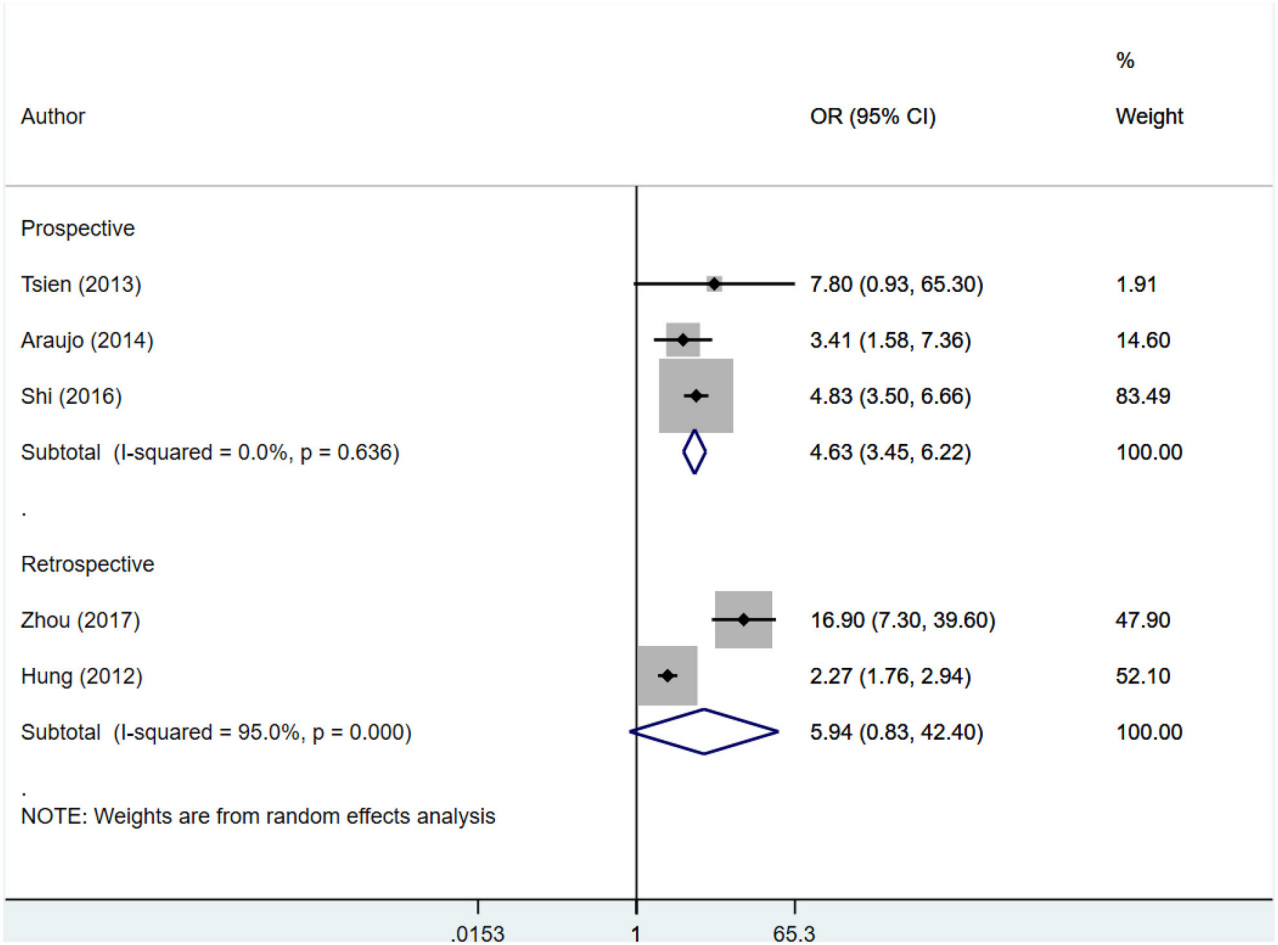
Subgroup analysis for association of acute kidney injury (AKI) with mortality at 1 year follow-up based on design of included studies.

An increased risk of in-hospital mortality (Prospective: OR 7.6, 95% CI: 5.4, 10.6; *N* = 8; *I*^2^ = 4.0%; Retrospective: OR 4.4, 95% CI: 2.6, 7.4; *N* = 6; *I*^2^ = 93.8%), 30-day mortality (Prospective: OR 4.7, 95% CI: 2.5, 9.1; *N* = 6; *I*^2^ = 89.9%; Retrospective: OR 4.9, 95% CI: 3.0, 7.9; *N* = 7; *I*^2^ = 86.6%) and 90-day mortality (Prospective: OR 4.4, 95% CI: 3.6, 5.3; *N* = 8; *I*^2^ = 0.0%; Retrospective: OR 4.0, 95% CI: 2.0, 8.2; *N* = 5; *I*^2^ = 84.8%) for patients with AKI was noted for both prospective and retrospective studies (Figures 3–5). Pooling of prospective studies showed an increased risk of mortality at 1 year follow-up (OR 4.6, 95% CI: 3.5, 6.2; *N* = 3; *I*^2^ = 0.0%). However, no increase was detected by pooling the retrospective studies (OR 5.9, 95% CI: 0.8, 42.4; *N* = 2; *I*^2^ = 95.0%) (Figure 6). A wide confidence intervals were noted, due to small number of studies. We noted low heterogeneity for prospective studies and a high heterogeneity for studies that used retrospective data. We next conducted a meta-regression using variables such as study design, geographical setting, sample size, and diagnostic criteria used for AKI and found that did not contribute to high heterogeneity (Supplementary Table 4).

## Discussion

The present review was conducted to document pooled evidence on the association of AKI with mortality in patients with cirrhosis. The key findings of our study were that the presence of AKI was associated with higher in-hospital mortality and mortality at 1 year follow-up, and that increasing risk of mortality correlated with an increase in the stage of AKI, in agreement with previous reviews [52,53]. Tariq et al. showed an increased risk of in-hospital mortality (OR 6.72) as well as mortality at 30 days (OR 3.37), 90 days (OR 4.43), and 12 months (OR 5.37) of follow-up among patients with cirrhosis and associated AKI [10]. A recent meta-analysis by Jiang et al. involving 8 studies (*n* = 3610 participants), investigated the impact of AKI on the outcome of acute-on-chronic liver failure [52]. The review found that the presence of AKI was associated with an increased risk of 1 month (OR 3.98) and 3 month mortality (OR 4.98). The review also found that the risk of mortality at 3 months follow-up increased with the increasing stage of AKI. Another review by Bai et al. aimed to understand the association between renal dysfunction or AKI and mortality among patients with cirrhosis and associated acute gastrointestinal bleeding [53]. A total of 17 studies were included. The pooled mortality was 47% in patients with AKI. The presence of renal dysfunction was also significantly associated with the increased risk of mortality (OR 4.92). The current meta-analysis acknowledged the previous reviews but aimed to use the most contemporary data to provide updated evidence on the effect of AKI on survival among cirrhotic patients. It also presented the risk of mortality based on different stages of AKI. Our results confirm findings, reported by previous reviews, thus further strengthening the evidence base that can be used in clinical practice.

Recent studies indicate that AKI in patients with cirrhosis is mostly caused by prerenal failure (32%), and acute tubular necrosis (35%) [54]. Patients with cirrhosis usually have consequent portal hypertension that results in splanchnic pooling of blood and leads to a decrease in the blood volume that is effectively circulating [8]. This presumably results in decreased perfusion to the kidneys and possibly sets the stage for the development of AKI. The onset of AKI can also be triggered by several other factors, such as blood volume loss, increased water loss due to the use of diuretics, nephrotoxic drugs, and acute stressors such as infections or sepsis [6,7,55,56]. Renal disease is associated with impaired immune function, characterized by abnormalities in the function of monocytes, neutrophils, and the associated leukopenia [57,58]. Evidence also indicates that AKI is associated with spontaneous bacterial peritonitis. It is possible that peritonitis which results from an intra-abdominal infection, leads to the translocation of bacteria outside of the gut into the blood circulation, and causes an intense inflammatory response that leads to subsequent renal damage and deterioration of renal function [59]. The findings of our review further support current clinical practice where an assessment and early detection of renal dysfunction in patients with cirrhosis is considered an important objective for therapeutic decision-making and for establishing prognosis. An interesting point to note is that the initial findings from some recent studies indicate that the use of terlipressin in patients with cirrhosis and acute upper gastrointestinal bleeding tends to improve renal function and prevent the occurrence of AKI [60]. Terlipressin, a vasopressin type 1 receptor agonist, is a commonly used drug in the treatment of acute variceal bleeding and hepatorenal syndrome that leads to decrease in serum creatinine, increase in glomerular filtration rate and natriuresis [61]. Further studies are still required to provide conclusive evidence on the effect of this drug in cirrhotic patients with ascites or acute upper gastrointestinal bleeding in the improvement of renal function and/or prevention of AKI. In all included studies, irrespective of the diagnostic criteria of AKI, serum creatinine was used for the diagnosis of AKI. Recent studies have suggested that cystatin C may be superior for predicting in-hospital mortality of cirrhotic patients with acute gastrointestinal bleeding [62]. Future studies should further explore the usefulness of the cystatin C-based prediction model.

There are some limitations of our meta-analysis. For most of the outcomes, there was a high degree of heterogeneity that could probably be due to methodological differences in the included studies. We did conduct a meta-regression to understand the factors leading to high heterogeneity such as study design, diagnostic criteria used for AKI, sample size, and geographic setting but found that the contribution of these factors to heterogeneity was not significant. The included studies were observational in design which made it impossible to adjust for important confounders and increased the possibility of selective reporting bias. Another important limitation is that among the sample of subjects included in the studies, roughly 5–35% had type 1 or 2 HRS. HRS is a potentially serious and life-threatening condition and the inclusion of subjects with HRS may have increased the observed risk of mortality.

## Conclusion

Our study concludes that the occurrence of AKI in patients with cirrhosis is associated with an increased risk of mortality. The findings call for close monitoring of patients with cirrhosis for any signs of AKI and subsequent careful management. This is particularly important as the study found an increased risk of mortality with the increase in stage of AKI. More prospective studies are required to provide detailed information on the clinical and prognostic outcomes of such patients.

## Supplementary Material

Supplemental Material

## Data Availability

The data that support the findings of this study are openly available in [PROSPERO] at [https://www.crd.york.ac.uk/prospero/display_record.php?ID=CRD42022324911], reference number [No CRD42022324911].

## References

[1] Cheemerla S, Balakrishnan M. Global epidemiology of chronic liver disease. Clin Liver Dis (Hoboken). 2021;17(5):365–370. 34136143 10.1002/cld.1061 PMC8177826

[2] Roth GA, Abate D, Abate KH, et al. Global, regional, and national age-sex-specific mortality for 282 causes of death in 195 countries and territories, 1980–2017: a systematic analysis for the Global Burden of Disease Study 2017. The Lancet. 2018;392(10159):1736–1788. 10.1016/S0140-6736(18)32203-7 PMC6227606 30496103

[3] Schuppan D, Afdhal NH. Liver cirrhosis. Lancet. 2008;371(9615):838–851. 18328931 10.1016/S0140-6736(08)60383-9 PMC2271178

[4] GBD 2017 Cirrhosis Collaborators. The global, regional, and national burden of cirrhosis by cause in 195 countries and territories, 1990-2017: a systematic analysis for the Global Burden of Disease Study 2017. Lancet Gastroenterol Hepatol. 2020;5:245–266. 31981519 10.1016/S2468-1253(19)30349-8 PMC7026710

[5] Bucsics T, Krones E. Renal dysfunction in cirrhosis: acute kidney injury and the hepatorenal syndrome. Gastroenterol Rep (Oxf). 2017;5(2):127–137. 28533910 10.1093/gastro/gox009 PMC5421450

[6] Gerbes AL. Liver cirrhosis and kidney. Dig Dis. 2016;34(4):387–390. 27170393 10.1159/000444553

[7] Garcia-Tsao G, Parikh CR, Viola A. Acute kidney injury in cirrhosis. Hepatology. 2008;48(6):2064–2077. 19003880 10.1002/hep.22605

[8] Ginès P, Schrier RW. Renal failure in cirrhosis. N Engl J Med. 2009;361(13):1279–1290. 19776409 10.1056/NEJMra0809139

[9] Rognant N, Lemoine S. Evaluation of renal function in patients with cirrhosis: where are we now? World J Gastroenterol. 2014;20(10):2533–2541. 24627589 10.3748/wjg.v20.i10.2533 PMC3949262

[10] Tariq R, Hadi Y, Chahal K, et al. Incidence, mortality and predictors of acute kidney injury in patients with cirrhosis: a systematic review and meta-analysis. J Clin Transl Hepatol. 2020;8(2):135–142. 32832393 10.14218/JCTH.2019.00060 PMC7438348

[11] Bellomo R, Ronco C, Kellum JA, et al. Acute renal failure - definition, outcome measures, animal models, fluid therapy and information technology needs: the Second International Consensus Conference of the Acute Dialysis Quality Initiative (ADQI) Group. Crit Care. 2004;8(4):R204–212. 15312219 10.1186/cc2872 PMC522841

[12] Mehta RL, Kellum JA, Shah SV, et al. Acute Kidney Injury Network: report of an initiative to improve outcomes in acute kidney injury. Crit Care. 2007;11(2):R31. 17331245 10.1186/cc5713 PMC2206446

[13] Khwaja A. KDIGO clinical practice guidelines for acute kidney injury. Nephron Clin Pract. 2012;120(4):c179–184. 22890468 10.1159/000339789

[14] Angeli P, Gines P, Wong F, et al. Diagnosis and management of acute kidney injury in patients with cirrhosis: revised consensus recommendations of the International Club of Ascites. Gut. 2015;64(4):531–537. 25631669 10.1136/gutjnl-2014-308874

[15] Lopes JA, Jorge S. The RIFLE and AKIN classifications for acute kidney injury: a critical and comprehensive review. Clin Kidney J. 2013;6(1):8–14. 27818745 10.1093/ckj/sfs160 PMC5094385

[16] PRISMA. Transparent reporting of systematic reviews and meta-analyses. [cited 2019 Jan 14]. Available from: http://www.prisma-statement.org/

[17] Wells G, Shea B, O’Connell D, et al. The Newcastle-Ottawa Scale (NOS) for assessing the quality of nonrandomized studies in meta- analysis. 2013. http://www.ohri.ca/programs/clinical_epidemiology/oxford.asp

[18] Higgins JP, Green S. Cochrane handbook for systematic reviews of interventions. Hoboken, NJ: Wiley; 2008.

[19] Egger M, Smith GD, Schneider M, et al. Bias in meta-analysis detected by a simple, graphical test. BMJ. 1997;315(7109):629–634. 9310563 10.1136/bmj.315.7109.629 PMC2127453

[20] Leão GS, de Mattos AA, Picon RV, et al. The prognostic impact of different stages of acute kidney injury in patients with decompensated cirrhosis: a prospective cohort study. Eur J Gastroenterol Hepatol. 2021;33(1S Suppl 1):e407–e412. 33731594 10.1097/MEG.0000000000002120

[21] Makar M, Reja D, Chouthai A, et al. The impact of acute kidney injury on mortality and clinical outcomes in patients with alcoholic cirrhosis in the USA. Eur J Gastroenterol Hepatol. 2021;33(6):905–910. 32976187 10.1097/MEG.0000000000001947

[22] Moga L, Robic M-A, Blasco-Perrin H, et al. Acute kidney injury in patients with cirrhosis: prospective longitudinal study in 405 patients. Clin Res Hepatol Gastroenterol. 2022;46(4):101822. 34718200 10.1016/j.clinre.2021.101822

[23] Arora MS, Kaushik R, Ahmad S, et al. Profile of acute kidney injury in patients with decompensated cirrhosis at a Tertiary-Care Center in Uttarakhand, India. Dig Dis. 2020;38(4):335–343. 31830752 10.1159/000504836

[24] Desai AP, Knapp SM, Orman ES, et al. Changing epidemiology and outcomes of acute kidney injury in hospitalized patients with cirrhosis - a US population-based study. J Hepatol. 2020;73(5):1092–1099. 32387698 10.1016/j.jhep.2020.04.043 PMC7994029

[25] Kumar U, Kumar R, Jha SK, et al. Short-term mortality in patients with cirrhosis of the liver and acute kidney injury: a prospective observational study. Indian J Gastroenterol. 2020;39(5):457–464. 33175368 10.1007/s12664-020-01086-z

[26] Vaz NF, da Cunha VNR, Cunha-Silva M, et al. Evolution of diagnostic criteria for acute kidney injury in patients with decompensated cirrhosis: a prospective study in a tertiary university hospital. Clin Res Hepatol Gastroenterol. 2020;44(4):551–563. 31427198 10.1016/j.clinre.2019.07.004

[27] Schacher FC, Mattos AA, Mulazzani CM, et al. Impact of acute kidney injury staging on prognosis of patients with cirrhosis. Arq Gastroenterol. 2020;57(3):244–248. 32935742 10.1590/S0004-2803.202000000-46

[28] Jo SK, Yang J, Hwang SM, et al. Role of biomarkers as predictors of acute kidney injury and mortality in decompensated cirrhosis. Sci Rep. 2019;9(1):14508. 31601879 10.1038/s41598-019-51053-8 PMC6787185

[29] Jaques DA, Spahr L, Berra G, et al. Biomarkers for acute kidney injury in decompensated cirrhosis: a prospective study. Nephrology (Carlton). 2019;24(2):170–180. 29369449 10.1111/nep.13226

[30] Marciano S, Mauro E, Dirchwolf M, et al. A dynamic definition of acute kidney injury does not improve prognosis assessment in acutely decompensated patients with cirrhosis. J Clin Exp Hepatol. 2017;7(2):135–143. 28663678 10.1016/j.jceh.2017.03.004 PMC5478969

[31] Nuthalapati A, Schluterman N, Khanna A, et al. Impact of acute kidney injury on mortality of patients hospitalized for complications of cirrhosis. J Clin Exp Hepatol. 2017;7(4):290–299. 29234192 10.1016/j.jceh.2017.05.004 PMC5720141

[32] Zhou F, Luo Q, Han L, et al. Evaluation of absolute serum creatinine changes in staging of cirrhosis-induced acute renal injury and its association with long-term outcomes. Kidney Blood Press Res. 2017;42(2):294–303. 28531894 10.1159/000477529

[33] Hsieh Y-C, Lee K-C, Chen P-H, et al. Acute kidney injury predicts mortality in cirrhotic patients with gastric variceal bleeding. J Gastroenterol Hepatol. 2017;32(11):1859–1866. 28271564 10.1111/jgh.13777

[34] Bıyık M, Ataseven H, Bıyık Z, et al. KDIGO (Kidney Disease: Improving Global Outcomes) criteria as a predictor of hospital mortality in cirrhotic patients. Turk J Gastroenterol. 2016;27(2):173–179. 27015622 10.5152/tjg.2016.15467

[35] Pan H-C, Chien Y-S, Jenq C-C, et al. Acute kidney injury classification for critically ill cirrhotic patients: a comparison of the KDIGO, AKIN, and RIFLE classifications. Sci Rep. 2016;6:23022. 26983372 10.1038/srep23022 PMC4794801

[36] Shi X, Zhu P, Yan G, et al. Clinical characteristics and long-term outcome of acute kidney injury in patients with HBV-related acute-on-chronic liver failure. J Viral Hepat. 2016;23(11):920–929. 27397610 10.1111/jvh.12566

[37] Jindal A, Bhadoria AS, Maiwall R, et al. Evaluation of acute kidney injury and its response to terlipressin in patients with acute-on-chronic liver failure. Liver Int. 2016;36(1):59–67. 26081914 10.1111/liv.12895

[38] Tandon P, James MT, Abraldes JG, et al. Relevance of new definitions to incidence and prognosis of acute kidney injury in hospitalized patients with cirrhosis: a retrospective population-based cohort study. PLoS One. 2016;11(8):e0160394. 27504876 10.1371/journal.pone.0160394 PMC4978466

[39] Bucsics T, Mandorfer M, Schwabl P, et al. Impact of acute kidney injury on prognosis of patients with liver cirrhosis and ascites: a retrospective cohort study. J Gastroenterol Hepatol. 2015;30(11):1657–1665. 25967931 10.1111/jgh.13002

[40] Angeli P, Rodríguez E, Piano S, et al. Acute kidney injury and acute-on-chronic liver failure classifications in prognosis assessment of patients with acute decompensation of cirrhosis. Gut. 2015;64(10):1616–1622. 25311034 10.1136/gutjnl-2014-307526

[41] Choi YJ, Kim JH, Koo JK, et al. Prevalence of renal dysfunction in patients with cirrhosis according to ADQI-IAC working party proposal. Clin Mol Hepatol. 2014;20(2):185–191. 25032185 10.3350/cmh.2014.20.2.185 PMC4099334

[42] de Araujo A, Alvares-da-Silva MR. Akin criteria as a predictor of mortality in cirrhotic patients after spontaneous bacterial peritonitis. Ann Hepatol. 2014;13(3):390–395. 24756016

[43] Piano S, Rosi S, Maresio G, et al. Evaluation of the Acute Kidney Injury Network criteria in hospitalized patients with cirrhosis and ascites. J Hepatol. 2013;59(3):482–489. 23665185 10.1016/j.jhep.2013.03.039

[44] Scott RA, Austin AS, Kolhe NV, et al. Acute kidney injury is independently associated with death in patients with cirrhosis. Frontline Gastroenterol. 2013;4(3):191–197. 24660054 10.1136/flgastro-2012-100291 PMC3955898

[45] Wong F, O'Leary JG, Reddy KR, et al. New consensus definition of acute kidney injury accurately predicts 30-day mortality in patients with cirrhosis and infection. Gastroenterology. 2013;145(6):1280–1288.e1. 23999172 10.1053/j.gastro.2013.08.051 PMC4418483

[46] Fagundes C, Barreto R, Guevara M, et al. A modified acute kidney injury classification for diagnosis and risk stratification of impairment of kidney function in cirrhosis. J Hepatol. 2013;59(3):474–481. 23669284 10.1016/j.jhep.2013.04.036

[47] Tsien CD, Rabie R, Wong F. Acute kidney injury in decompensated cirrhosis. Gut. 2013;62(1):131–137. 22637695 10.1136/gutjnl-2011-301255

[48] Hung T-H, Tsai C-C, Hsieh Y-H, et al. Effect of renal impairment on mortality of patients with cirrhosis and spontaneous bacterial peritonitis. Clin Gastroenterol Hepatol. 2012;10(6):677–681. 22391345 10.1016/j.cgh.2012.02.026

[49] Warner NS, Cuthbert JA, Bhore R, et al. Acute kidney injury and chronic kidney disease in hospitalized patients with cirrhosis. J Investig Med. 2011;59(8):1244–1251. 10.2130/JIM.0b013e3182321471 21941210

[50] Cholongitas E, Senzolo M, Patch D, et al. Cirrhotics admitted to intensive care unit: the impact of acute renal failure on mortality. Eur J Gastroenterol Hepatol. 2009;21(7):744–750. 10.1097/MEG.0b013e328308bb9c. 20160527

[51] Du Cheyron D, Bouchet B, Parienti J-J, et al. The attributable mortality of acute renal failure in critically ill patients with liver cirrhosis. Intensive Care Med. 2005;31(12):1693–1699. 16244877 10.1007/s00134-005-2842-7

[52] Jiang W, Hu Y, Sun Y, et al. Prevalence and short-term outcome of acute kidney injury in patients with acute-on-chronic liver failure: a meta-analysis. J Viral Hepat. 2020;27(8):810–817. 32141141 10.1111/jvh.13287

[53] Bai Z, Primignani M, Guo X, et al. Incidence and mortality of renal dysfunction in cirrhotic patients with acute gastrointestinal bleeding: a systematic review and meta-analysis. Expert Rev Gastroenterol Hepatol. 2019;13(12):1181–1188. 31736376 10.1080/17474124.2019.1694904

[54] Muciño-Bermejo J, Carrillo-Esper R, Uribe M, et al. Acute kidney injury in critically ill cirrhotic patients: a review. Ann Hepatol. 2012;11(3):301–310. 22481447

[55] Gessolo Lins PR, Carvalho Padilha WS, Magalhaes Giradin Pimentel CF, et al. Risk factors, mortality and acute kidney injury outcomes in cirrhotic patients in the emergency department. BMC Nephrol. 2018;19(1):277. 30342475 10.1186/s12882-018-1061-8 PMC6196026

[56] Duah A, Duah F, Ampofo-Boobi D, et al. Acute kidney injury in patients with liver cirrhosis: prevalence, predictors, and in-hospital mortality at a district hospital in Ghana. Biomed Res Int. 2022;2022:4589767. 35237687 10.1155/2022/4589767 PMC8885249

[57] Singbartl K, Formeck CL, Kellum JA. Kidney-immune system crosstalk in AKI. Semin Nephrol. 2019;39(1):96–106. 30606411 10.1016/j.semnephrol.2018.10.007

[58] LaFavers K. Disruption of kidney-immune system crosstalk in sepsis with acute kidney injury: lessons learned from animal models and their application to human health. IJMS. 2022;23(3):1702. 35163625 10.3390/ijms23031702 PMC8835938

[59] Peng Q, Zhang L, Ai Y, et al. Epidemiology of acute kidney injury in intensive care septic patients based on the KDIGO guidelines. Chin Med J (Engl). 2014;127(10):1820–1826. 24824238

[60] An Y, Bai Z, Guo X, et al. Effect of terlipressin on renal function in liver cirrhosis with ascites: a pilot study. J Clin Exp Hepatol. 2020;10(6):643–645. 33311900 10.1016/j.jceh.2020.05.002 PMC7720021

[61] Zhang J, Liu J, Wu Y, et al. Effect of terlipressin on renal function in cirrhotic patients with acute upper gastrointestinal bleeding. Ann Transl Med. 2020;8(6):340. 32355784 10.21037/atm.2020.02.135 PMC7186671

[62] Hong C, Zhu Q, Li Y, et al. Acute kidney injury defined by cystatin C may be superior for predicting the outcomes of liver cirrhosis with acute gastrointestinal bleeding. Ren Fail. 2022;44(1):398–406. 35225149 10.1080/0886022X.2022.2039193 PMC8890530

