# Supplementary material for: Impact of acute kidney injury on the risk of mortality in patients with cirrhosis: a systematic review and meta-analysis
Source: Ren Fail. 2022 Nov 15;44(1):1–14. doi: 10.1080/0886022X.2022.2142137 (PMC9673785; doi:10.1080/0886022X.2022.2142137)

SUPPLEMENTARY DOCUMENT

Box 1. Search strategy used to identify potential studies for inclusion in the meta-analysis

Search strategy in PubMed

#1 (cirrhosis OR liver disease OR hepatic cirrhosis OR liver cirrhosis)
#2 (Kidney injury OR acute kidney injury OR AKI OR renal insufficiency OR acute renal insufficiency)
#3 (complications OR clinical outcomes OR mortality)
#4 (#1 AND #2 AND #3)
#5 (Addresses[ptyp] OR Autobiography[ptyp] OR Bibliography[ptyp] OR Biography[ptyp] OR pubmed books[filter] OR Case Reports[ptyp] OR Congresses[ptyp] OR Consensus Development Conference[ptyp] OR Directory[ptyp] OR Duplicate Publication[ptyp] OR Editorial[ptyp] OR Systematic reviews OR Meta analysis OR Festschrift[ptyp] OR Guideline[ptyp] OR In Vitro[ptyp] OR Interview[ptyp] OR Lectures [ptyp] OR Legal Cases[ptyp] OR News[ptyp] OR Newspaper Article[ptyp] OR Personal Narratives [ptyp] OR Portraits[ptyp] OR Retracted Publication[ptyp] OR Twin Study[ptyp] OR Video-Audio Media[ptyp])
#6 (#4 NOT #5)

Search strategy in Embase
(cirrhosis or liver disease or hepatic cirrhosis or liver cirrhosis or liver pathology/) and (Kidney* or Kidney injury/ or AKI or renal* or renal insufficiency or acute renal*) AND 'mortality':ti,ab,kw or “complications’; ti,ab,kw or “death”: ti,ab,kw or “survival” : ti,ab,kw

Search strategy in Scopus

#1 (liver or hepatic) and (cirrho *) OR TITLE-ABS-KEY (cirrho*)
#2 (Acute kidney * or AKI or renal insuff*) OR TITLE-ABS-KEY (acute renal*)
#3 (survival or mortality or compli* or death) OR TITLE-ABS-KEY (“mortality”)

#1 and #2 and #3

Supplementary table 1. Author's judgements about study quality using the adapted Ottawa-Newcastle Risk of Bias Assessment tool

	Moga et al	Sang et al	Arora et al	Desai et al	Kumar et al	Leao et al	Makar et al	Vaz et al	Schacher et al	Biyik et al	Choi et al	Araujo et al	Cheyron et al
Representativeness/appropriateness of participant selection<br>Random or consecutive recruitment=Y<br>Convenience sample=N<br>Not reported or unclear	Y	Y	Y	Y	Y	Y	Y	Y	Y	Y	Y	Y	Y
Control for baseline differences in cohorts<br>Similarity of groups at baseline or adjustment in analyses=Y<br>No attempt to control or adjust=N<br>Not reported=NR	Y	Y	N	Y	Y	Y	Y	Y	Y	Y	N	Y	Y
Loss to follow-up<br>Explanation provided for loss of participants and/or intention to treat=Y<br>No explanation =N	Y	Y	Y	Y	Y	Y	Y	Y	Y	Y	Y	Y	Y
Masking of exposure to outcomes assessor<br>Description of masking=Y<br>No masking or no description =N	Y	Y	Y	Y	Y	Y	Y	Y	Y	Y	Y	Y	Y
Ascertainment of condition<br>Description of ascertainment/diagnostic criteria=Y<br>No description or patient self-report=N	Y	Y	Y	Y	N	Y	Y	N	Y	Y	Y	Y	Y
Documentation of other treatment modalities<br>Documentation=Y<br>No documentation=N	Y	Y	Y	N	Y	Y	Y	Y	Y	Y	Y	Y	Y

Extent to which valid outcomes are described<br>Adequate description of outcome=Y<br>Insufficient detail regarding outcome or follow-up time=N	Y	Y	Y	Y	Y	Y	Y	Y	Y	Y	Y	Y	Y
Prespecification of harms, mode of harms collection<br>Description of a list of harms assessed or monitoring=Y<br>No such description or passive harms collection=N<br>No adverse events reported=NA	Y	Y	Y	Y	Y	Y	Y	N	Y	N	Y	Y	N
Financial Conflict of interest (COI)<br>Funding source reported=Y<br>Funding source not reported=N	N	Y	Y	N	N	Y	Y	Y	Y	Y	N	Y	Y

Supplementary table 2. Author's judgements about study quality using the adapted Ottawa-Newcastle Risk of Bias Assessment tool

	Jaques et al	Piano et al	Scott et al	Warner et al	Cholongitas et al	Pan et al	Bucsics et al	Hung et al	Jindal et al	Marciano et al	Nuthalapti et al	Shi et al	Wong et al
Representativeness/appropriateness of participant selection<br>Random or consecutive recruitment=Y<br>Convenience sample=N<br>Not reported or unclear	Y	Y	Y	Y	Y	Y	Y	Y	Y	Y	Y	Y	Y
Control for baseline differences in cohorts<br>Similarity of groups at baseline or adjustment in analyses=Y<br>No attempt to control or adjust=N<br>Not reported=NR	Y	Y	N	Y	Y	Y	N	Y	Y	Y	Y	Y	Y
Loss to follow-up<br>Explanation provided for loss of participants and/or intention to treat=Y<br>No explanation =N	Y	Y	N	Y	Y	Y	Y	Y	Y	Y	Y	Y	Y
Masking of exposure to outcomes assessor<br>Description of masking=Y<br>No masking or no description =N	Y	Y	Y	Y	Y	Y	Y	Y	Y	Y	Y	Y	Y
Ascertainment of condition<br>Description of ascertainment/diagnostic criteria=Y<br>No description or patient self-report=N	Y	Y	Y	Y	N	Y	Y	N	Y	Y	Y	Y	Y
Documentation of other treatment modalities<br>Documentation=Y<br>No documentation=N	Y	Y	Y	N	Y	Y	Y	Y	Y	Y	Y	Y	Y

Extent to which valid outcomes are described<br>Adequate description of outcome=Y<br>Insufficient detail regarding outcome or follow-up time=N	Y	Y	Y	Y	Y	Y	Y	Y	Y	Y	Y	Y	Y
Prespecification of harms, mode of harms collection<br>Description of a list of harms assessed or monitoring=Y<br>No such description or passive harms collection=N<br>No adverse events reported=NA	Y	Y	Y	Y	Y	N	N	Y	Y	Y	Y	Y	N
Financial Conflict of interest (COI)<br>Funding source reported=Y<br>Funding source not reported=N	Y	N	Y	N	Y	Y	Y	N	Y	Y	Y	Y	Y

Supplementary table 3. Author's judgements about study quality using the adapted Ottawa-Newcastle Risk of Bias Assessment tool

	Fagundes et al	Hsieh et al	Tandon et al	Tsien et al	Zhou et al	Angeli et al
Representativeness/appropriateness of participant selection<br>Random or consecutive recruitment=Y<br>Convenience sample=N<br>Not reported or unclear	Y	Y	Y	Y	Y	Y
Control for baseline differences in cohorts<br>Similarity of groups at baseline or adjustment in analyses=Y<br>No attempt to control or adjust=N<br>Not reported=NR	Y	Y	Y	Y	Y	Y
Loss to follow-up<br>Explanation provided for loss of participants and/or intention to treat=Y<br>No explanation =N	Y	Y	Y	Y	N	Y
Masking of exposure to outcomes assessor<br>Description of masking=Y<br>No masking or no description =N	Y	Y	Y	Y	Y	Y
Ascertainment of condition<br>Description of ascertainment/diagnostic criteria=Y<br>No description or patient self-report=N	Y	Y	Y	Y	Y	Y
Documentation of other treatment modalities<br>Documentation=Y<br>No documentation=N	Y	Y	Y	N	Y	Y
Extent to which valid outcomes are described<br>Adequate description of outcome=Y<br>Insufficient detail regarding outcome or follow-up time=N	Y	Y	Y	Y	Y	Y
Prespecification of harms, mode of harms collection<br>Description of a list of harms assessed or monitoring=Y<br>No such description or passive harms collection=N<br>No adverse events reported=NA	Y	Y	Y	Y	N	Y
Financial Conflict of interest (COI)<br>Funding source reported=Y<br>Funding source not reported=N	Y	Y	N	Y	Y	Y

Supplementary table 4. Findings of meta-regression

	Exp (b)	SE	95% CI	P-value
**Mortality (in-hospital)**				
AKI criteria	0.65	0.22	0.30, 1.40	0.232
Study design (retrospective or prospective)	0.53	0.21	0.22, 1.30	0.148
Study setting (developed or developing country)	1.52	1.28	0.24, 9.60	0.633
Sample size	0.72	0.35	0.25, 2.10	0.522
**Mortality (30-day)**				
AKI criteria	0.92	0.27	0.47, 1.80	0.773
Study design (retrospective or prospective)	1.06	0.41	0.45, 2.49	0.884
Study setting (developed or developing country)	0.64	0.23	0.29, 1.41	0.237
Sample size (<500 and ≥500)	0.80	0.32	0.33, 1.93	0.591
**Mortality (90-day)**				
AKI criteria	0.89	0.16	0.60, 1.34	0.550
Study design (retrospective or prospective)	0.98	0.30	0.50, 1.90	0.939
Study setting (developed or developing country)	1.03	0.31	0.53, 1.98	0.936
Sample size (<500 and ≥500)	1.39	0.39	0.75, 2.56	0.263
**Mortality (1-year)**				
AKI criteria	0.57	0.38	0.03, 9.85	0.483
Study design (retrospective or prospective)	1.22	1.06	0.08, 19.35	0.837
Study setting (developed or developing country)	2.10	1.57	0.20, 22.5	0.392
Sample size (<500 and ≥500)	0.44	0.31	0.05, 4.25	0.333

Supplementary Figure 1. Funnel plot for in-hospital mortality

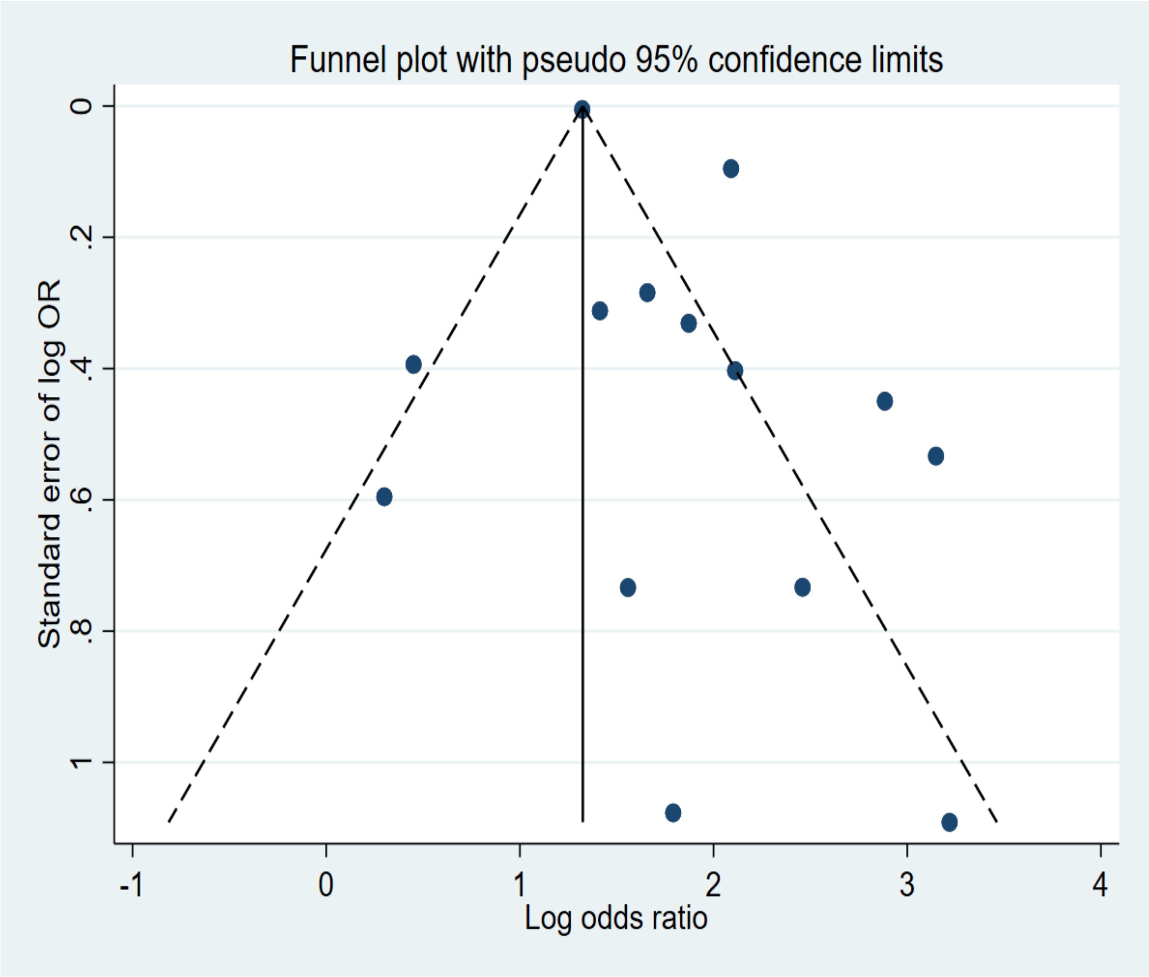

Supplementary Figure 2. Funnel plot for 30-day mortality

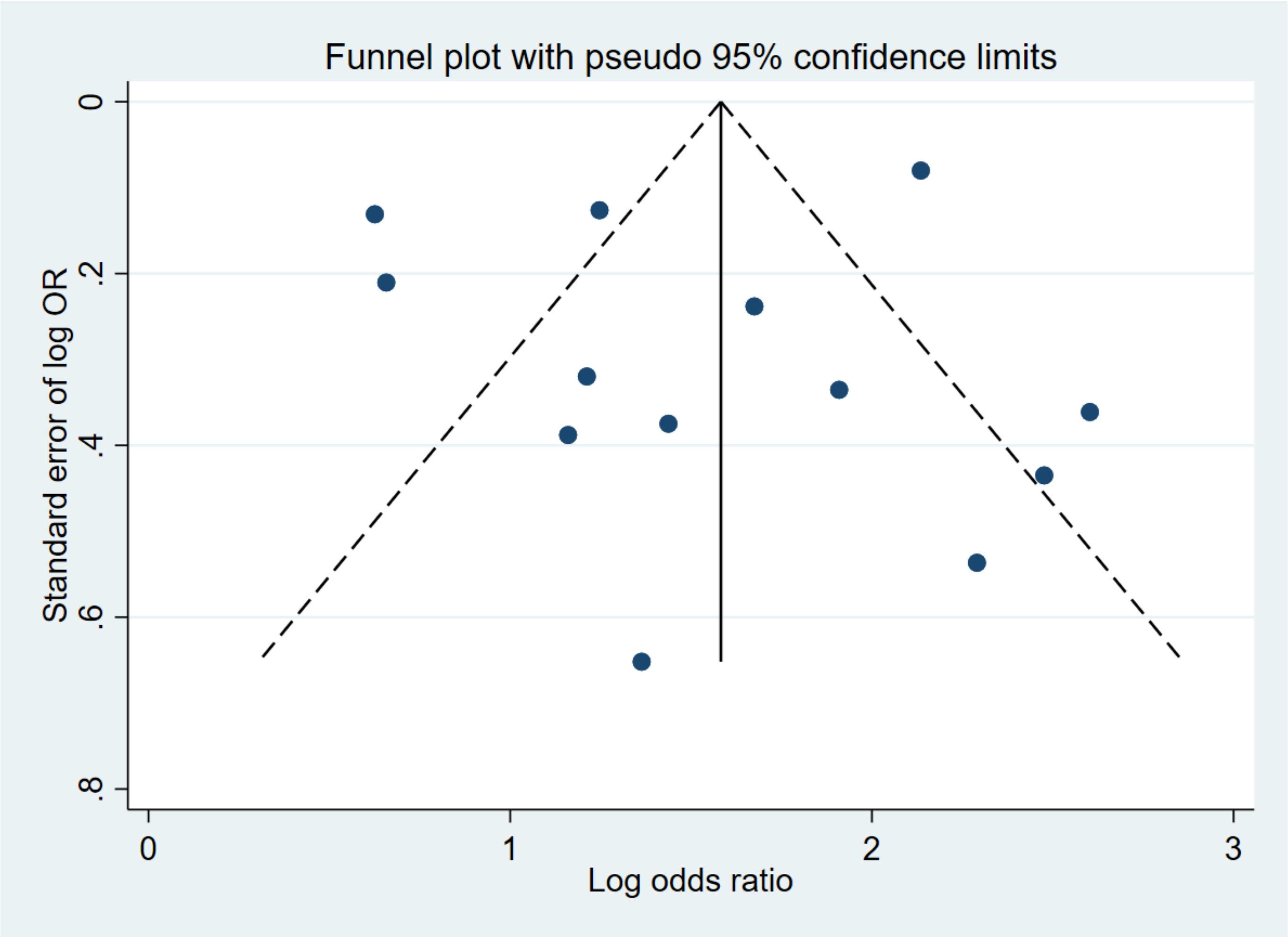

Supplementary Figure 3. Funnel plot for 90-day mortality

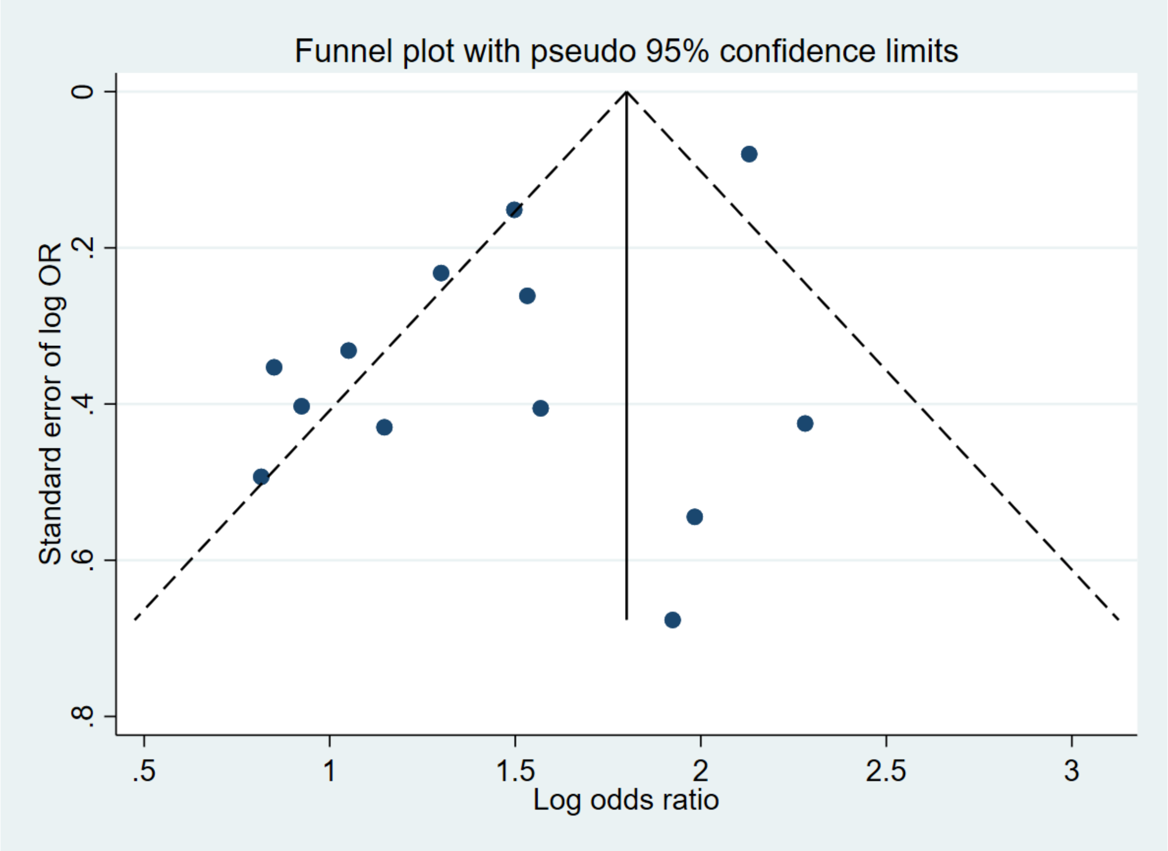

Supplementary Figure 4. Funnel plot for mortality at 1 year follow up

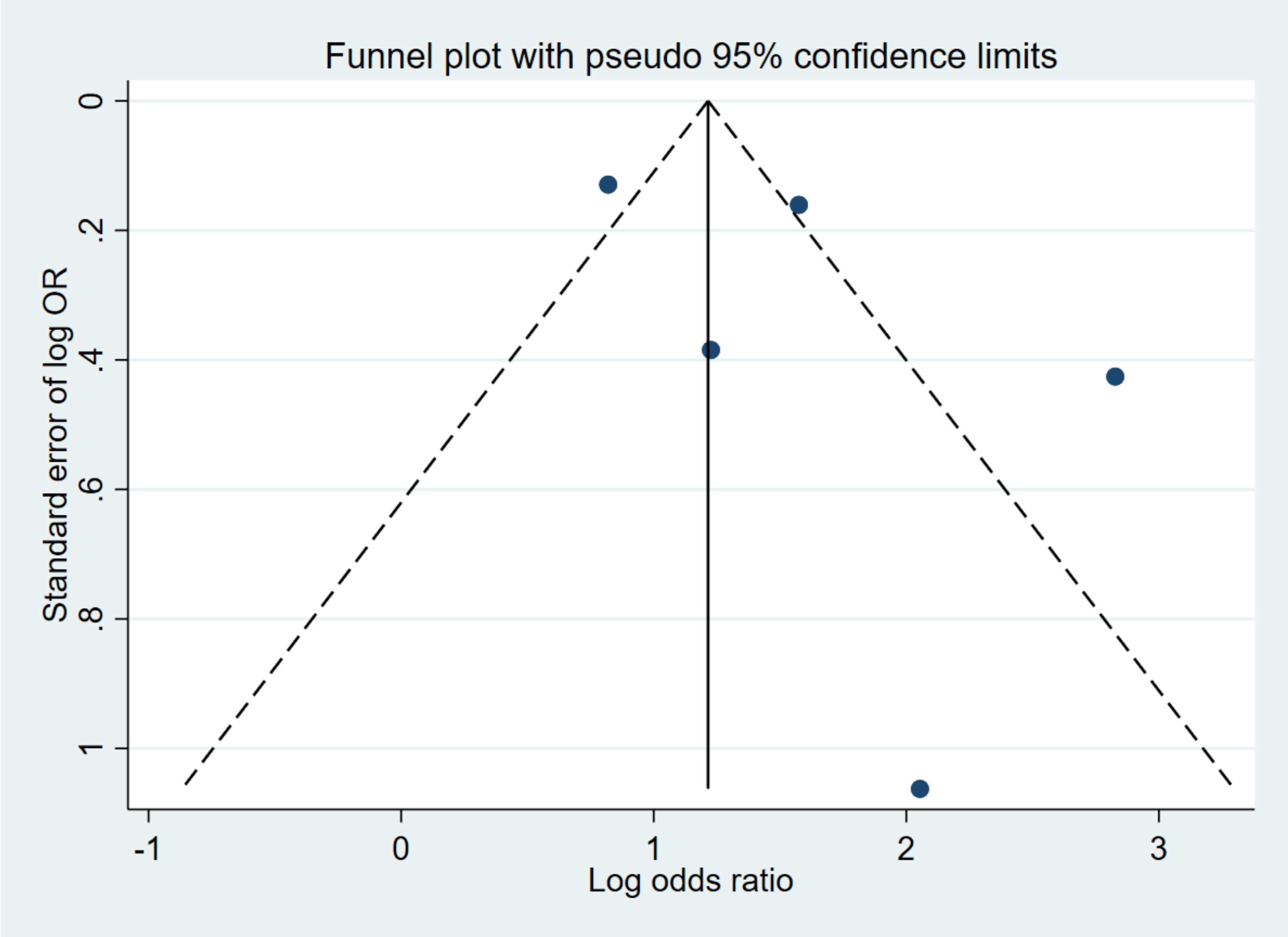

Supplementary figure 5: Risk of in-hospital mortality stratified by the criteria of AKI used in the included studies

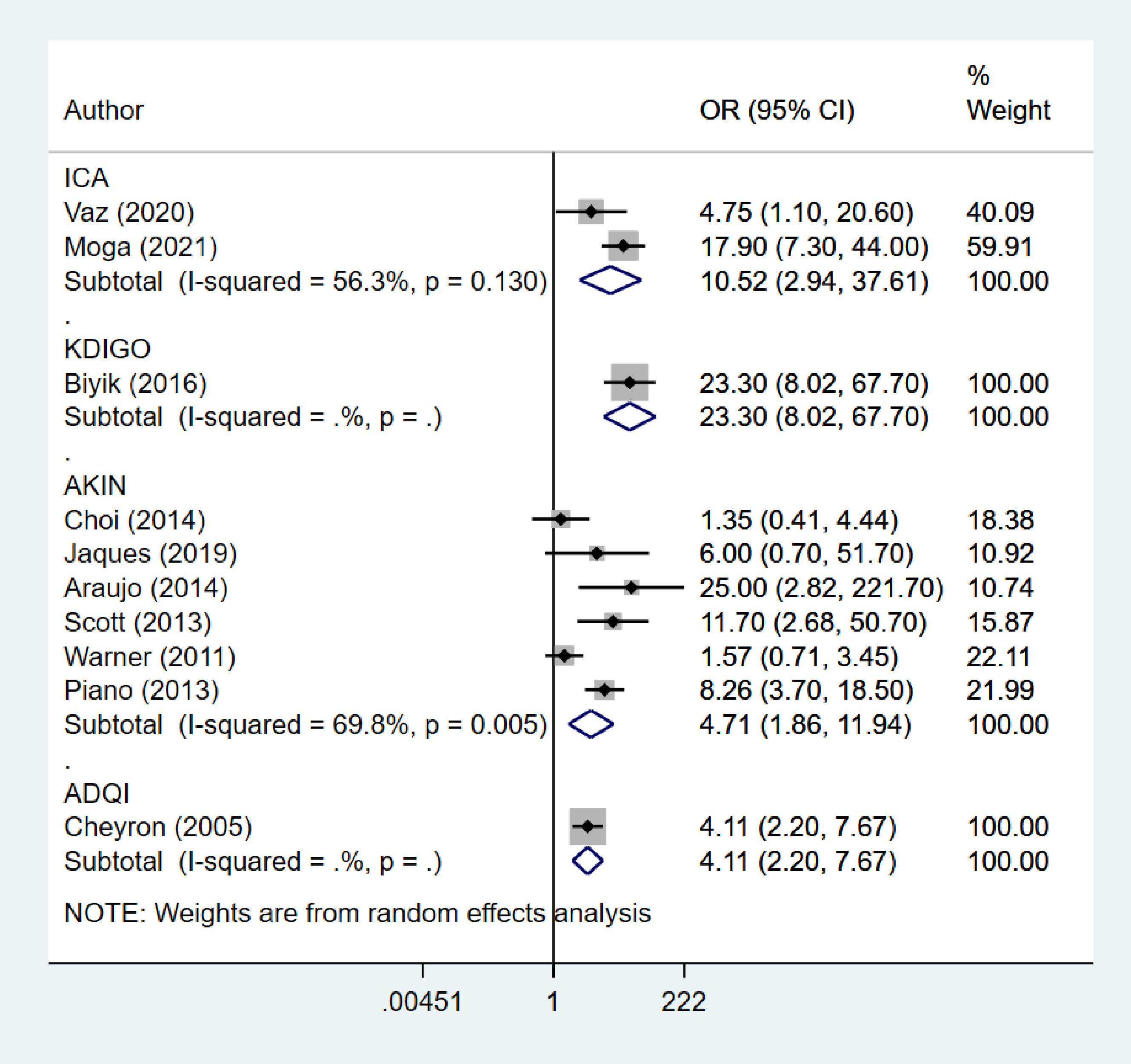

Supplementary figure 6: Risk of 30-day mortality stratified by the criteria of AKI used in the included studies

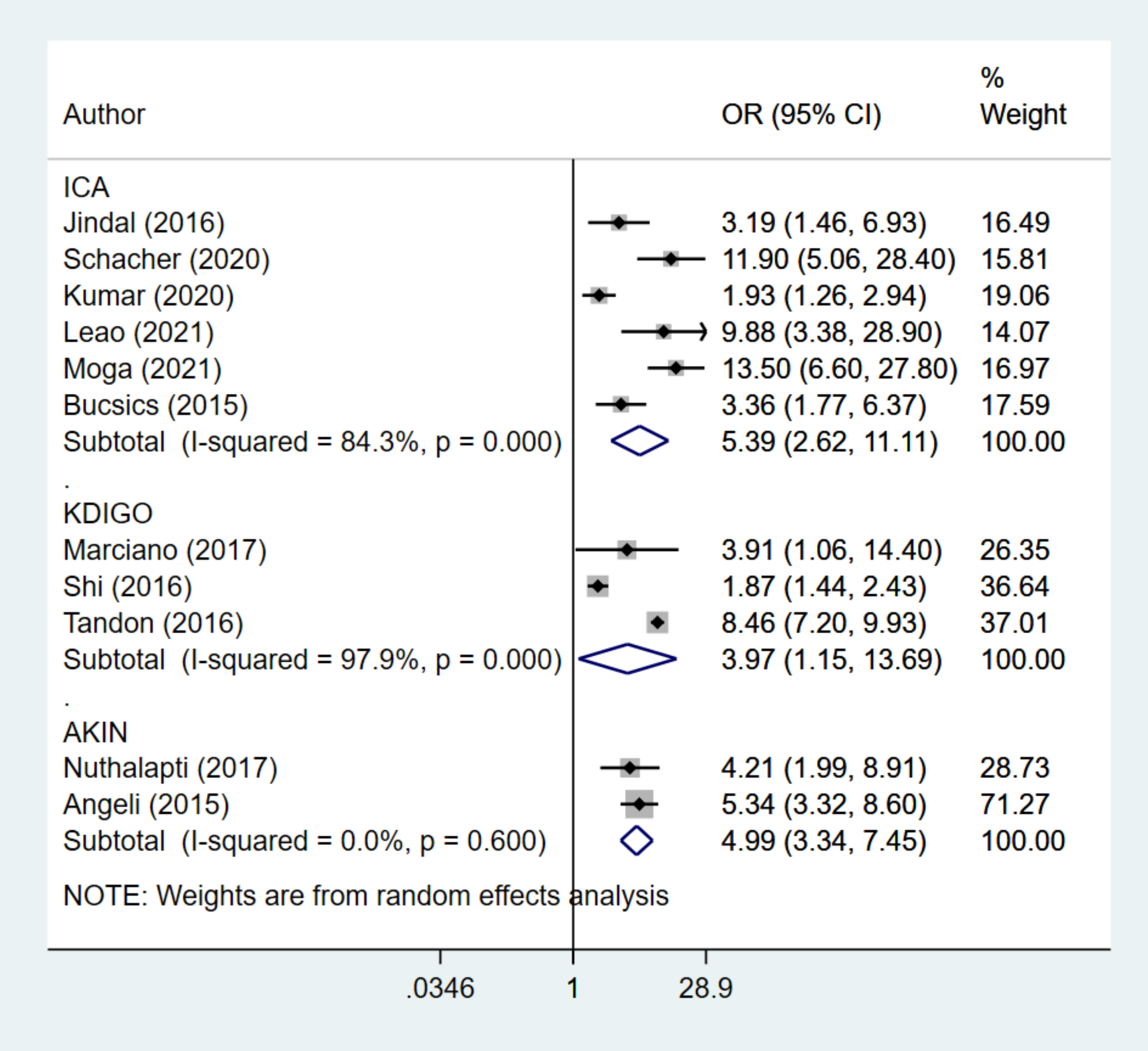

Supplementary figure 7: Risk of 90-day mortality stratified by the criteria of AKI used in the included studies

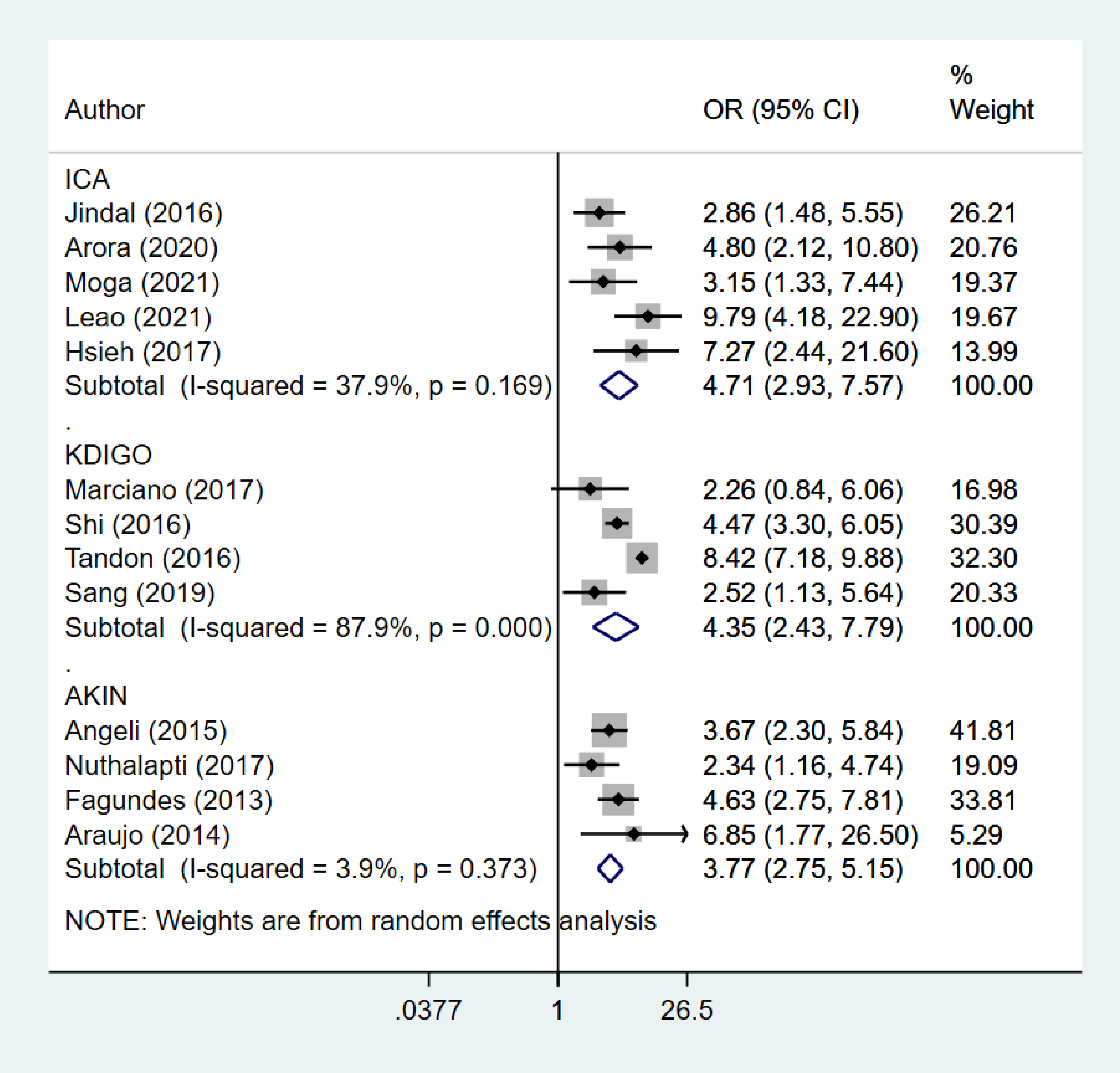

Supplementary figure 8: Risk of mortality at 1-year follow up, stratified by the criteria of AKI used in the included studies

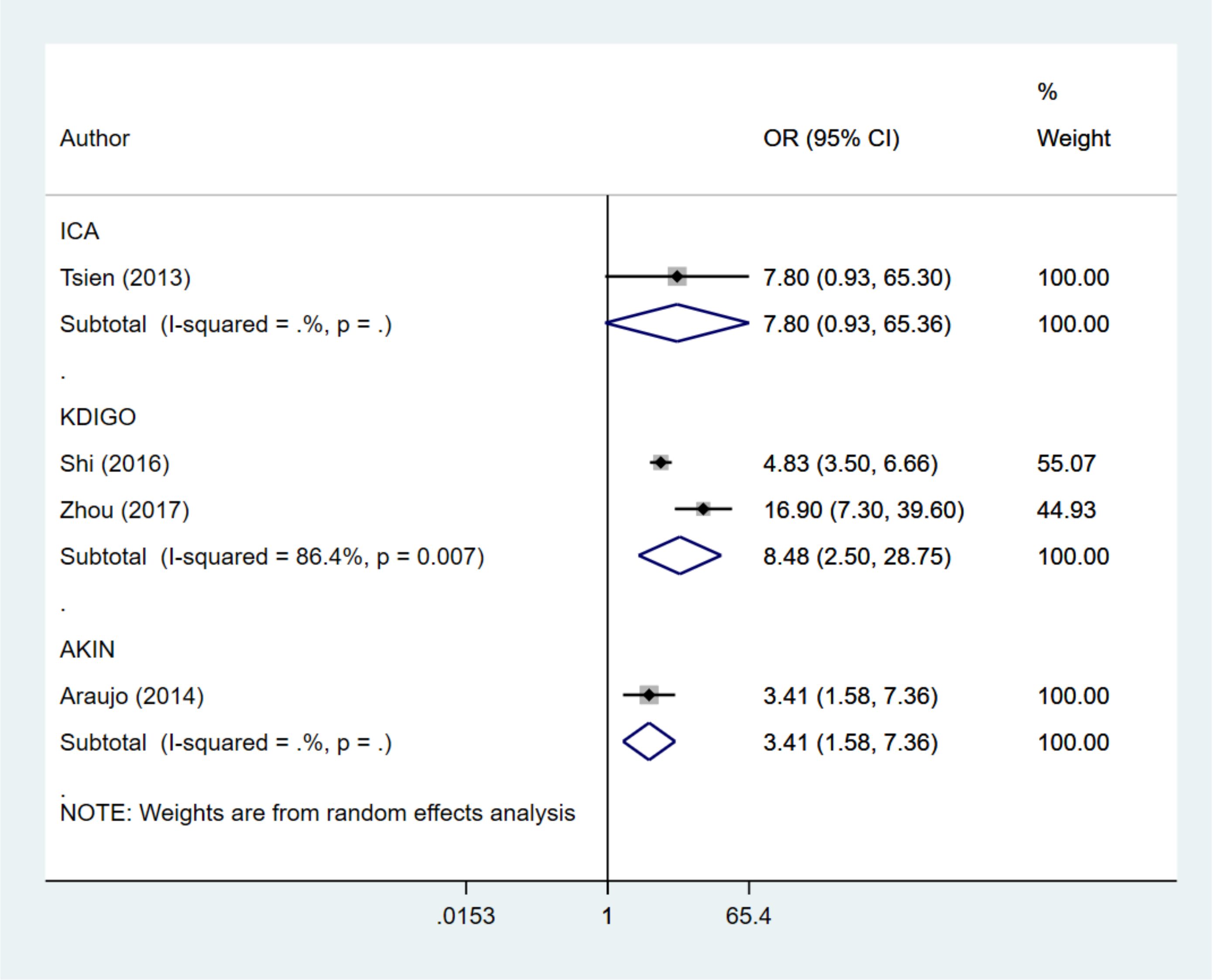

Supplementary figure 9: Risk of in-hospital mortality, stratified by stage of AKI

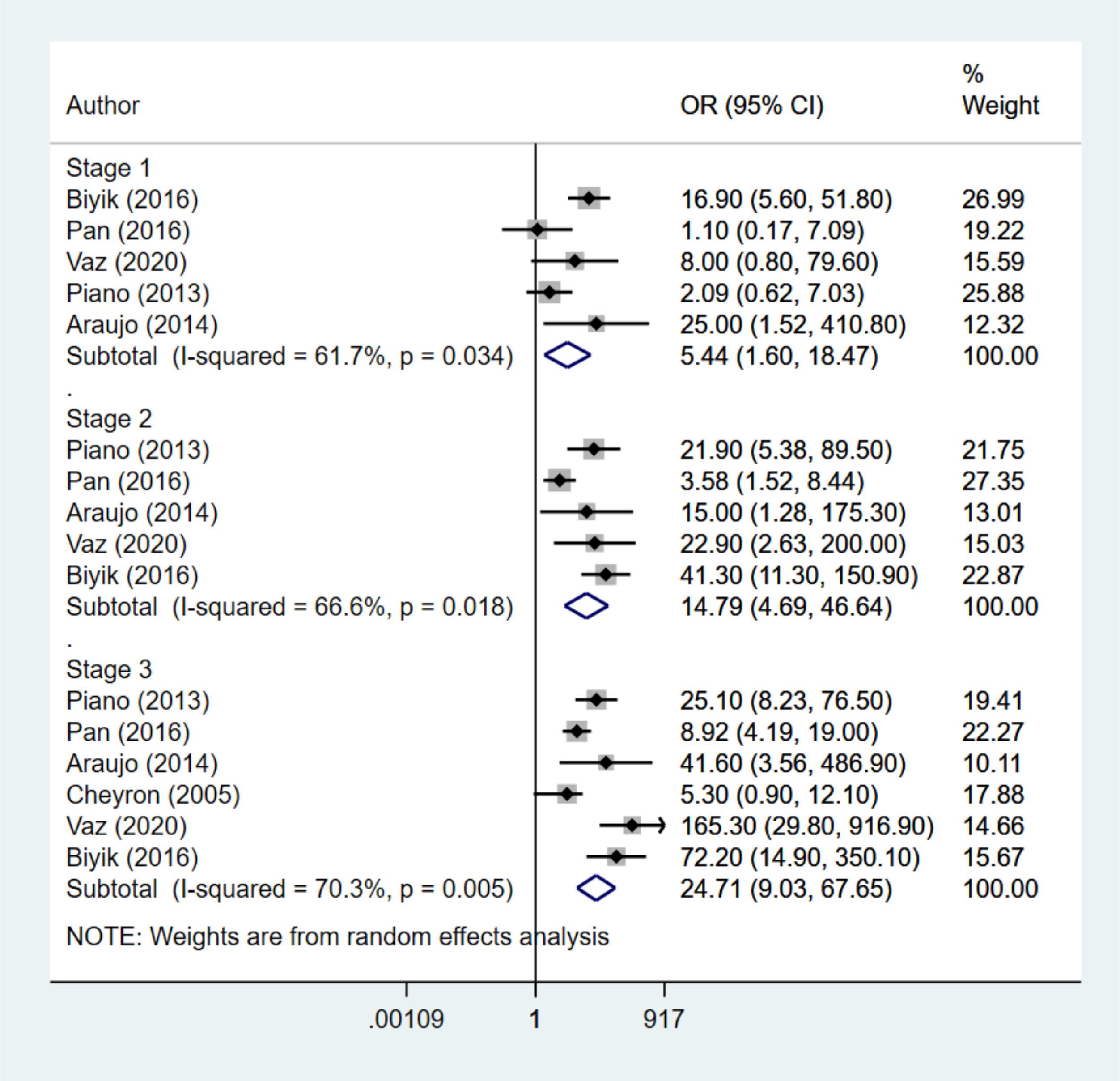

Supplementary figure 10: Risk of 30-day mortality, stratified by stage of AKI

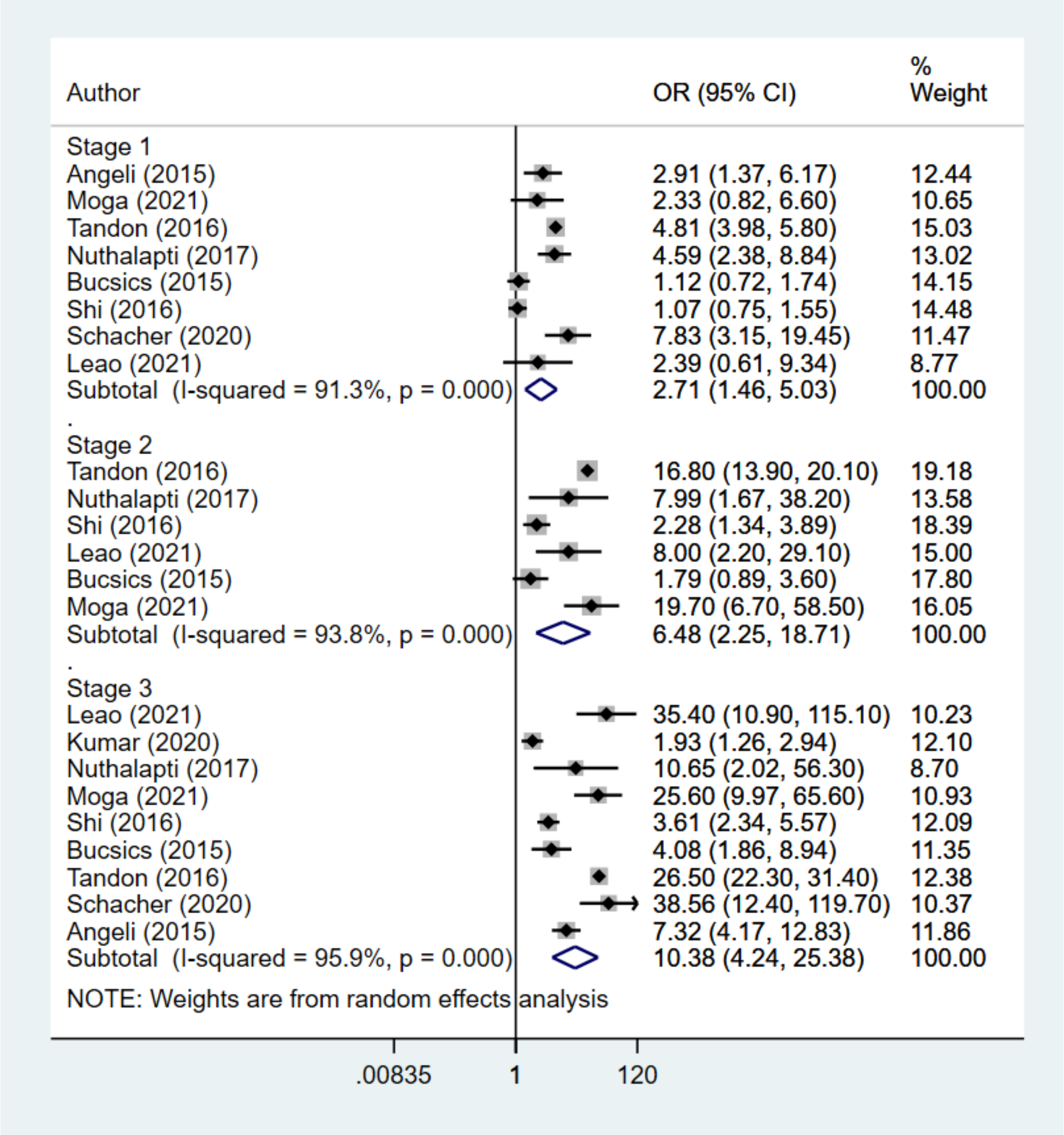

Supplementary figure 11: Risk of 90-day mortality, stratified by stage of AKI

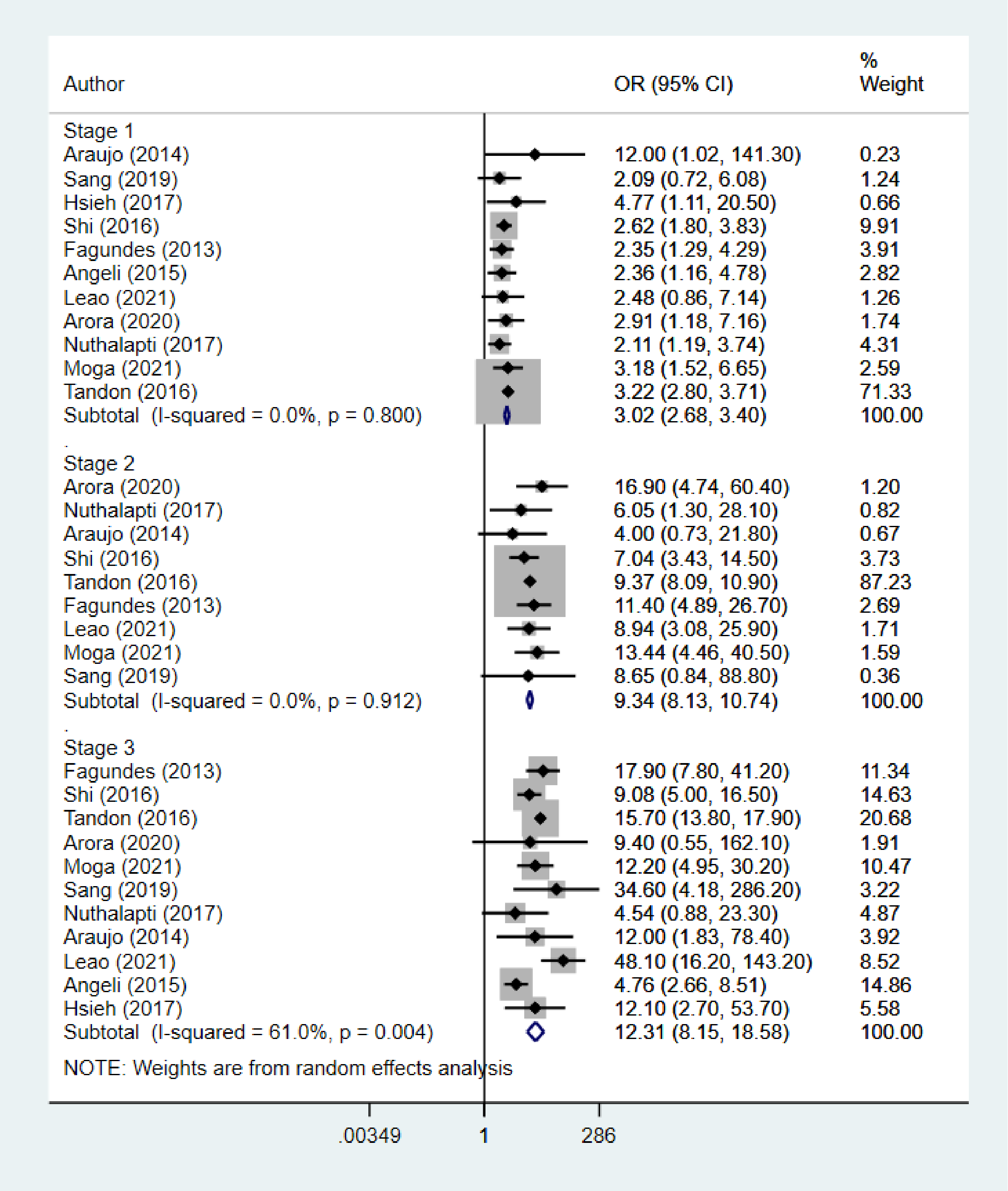

Supplementary figure 12: Risk of mortality at 1-year follow up, stratified by stage of AKI

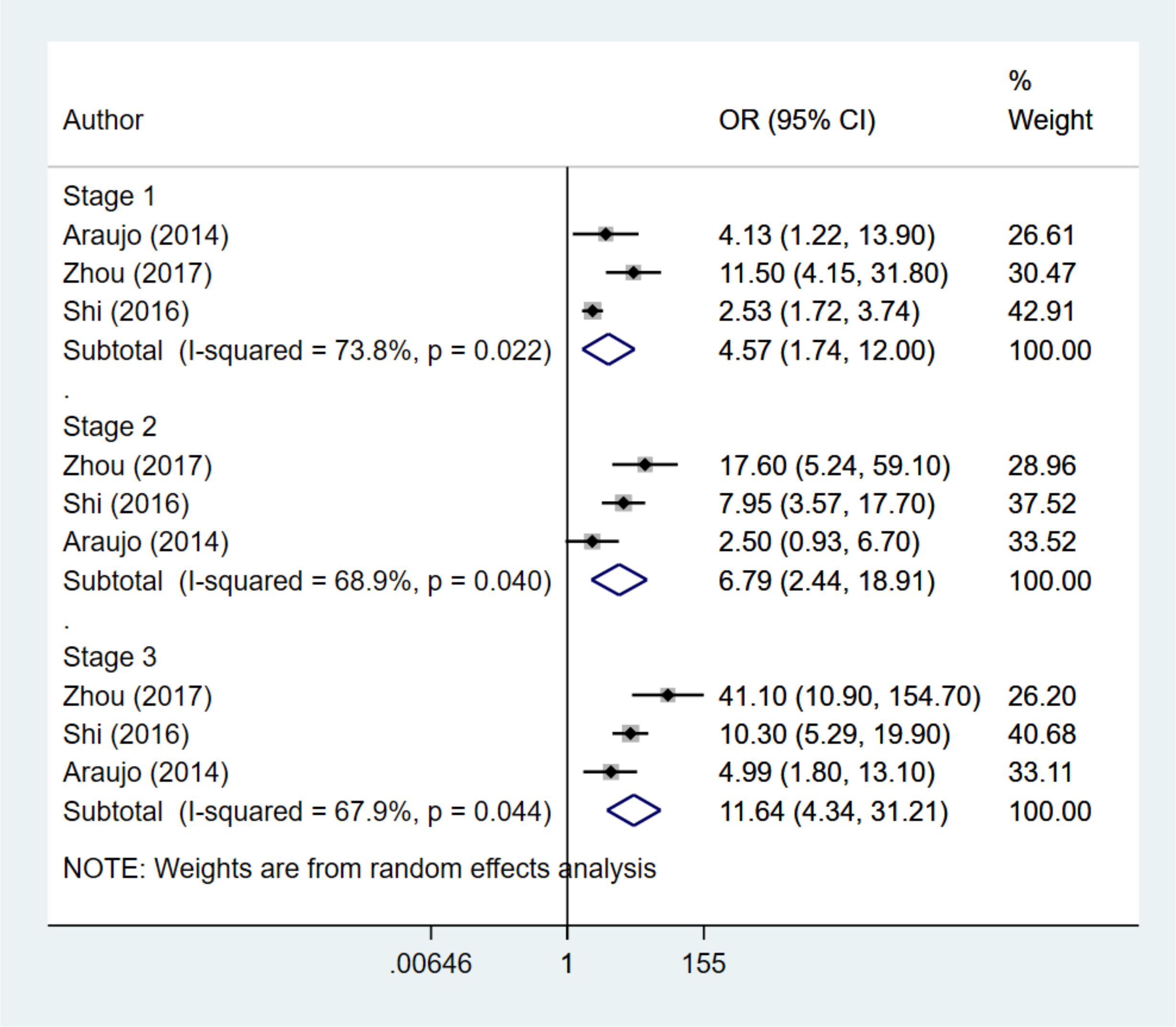

Supplement: Supplemental Material [file IRNF_A_2142137_SM8104.pdf]
